# Development and characterization of *Escherichia coli* triple reporter strains for investigation of population heterogeneity in bioprocesses

**DOI:** 10.1186/s12934-020-1283-x

**Published:** 2020-01-28

**Authors:** Anna-Lena Heins, Jan Reyelt, Marlen Schmidt, Harald Kranz, Dirk Weuster-Botz

**Affiliations:** 1 Technical University of Munich, Institute of Biochemical Engineering, Boltzmannstr. 15, 85748 Garching, Germany; 2 Gene Bridges GmbH, Im Neuenheimer Feld 584, 69120 Heidelberg, Germany

**Keywords:** Reporter strain, Single cell physiology, Population heterogeneity, Bioprocess, Noise in gene expression

## Abstract

**Background:**

Today there is an increasing demand for high yielding robust and cost efficient biotechnological production processes. Although cells in these processes originate from isogenic cultures, heterogeneity induced by intrinsic and extrinsic influences is omnipresent. To increase understanding of this mechanistically poorly understood phenomenon, advanced tools that provide insights into single cell physiology are needed.

**Results:**

Two *Escherichia coli* triple reporter strains have been designed based on the industrially relevant production host *E. coli* BL21(DE3) and a modified version thereof, *E. coli* T7E2. The strains carry three different fluorescence proteins chromosomally integrated. Single cell growth is followed with EmeraldGFP (EmGFP)-expression together with the ribosomal promoter *rrnB*. General stress response of single cells is monitored by expression of sigma factor *rpoS* with mStrawberry, whereas expression of the *nar*-operon together with TagRFP657 gives information about oxygen limitation of single cells. First, the strains were characterized in batch operated stirred-tank bioreactors in comparison to wildtype *E. coli* BL21(DE3). Afterwards, applicability of the triple reporter strains for investigation of population heterogeneity in bioprocesses was demonstrated in continuous processes in stirred-tank bioreactors at different growth rates and in response to glucose and oxygen perturbation simulating gradients on industrial scale. Population and single cell level physiology was monitored evaluating general physiology and flow cytometry analysis of fluorescence distributions of the triple reporter strains. Although both triple reporter strains reflected physiological changes that were expected based on the expression characteristics of the marker proteins, the triple reporter strain based on *E. coli* T7E2 showed higher sensitivity in response to environmental changes. For both strains, noise in gene expression was observed during transition from phases of non-growth to growth. Apparently, under some process conditions, e.g. the stationary phase in batch cultures, the fluorescence response of EmGFP and mStrawberry is preserved, whereas TagRFP657 showed a distinct response.

**Conclusions:**

Single cell growth, general stress response and oxygen limitation of single cells could be followed using the two triple reporter strains developed in this study. They represent valuable tools to study population heterogeneity in bioprocesses significantly increasing the level of information compared to the use of single reporter strains.

## Background

Today there is a constantly increasing interest in biotechnological products such as pharmaceuticals or products for dairy or food industry [1]. To fulfil the demand of robust high-yielding bioprocesses for a biotechnological product of interest, ideally, all cells should exhibit the same constant and high productivity. However, although single cells in a population in bioreactors on an industrial scale originate from isogenic cultures, they can be metabolically diverse and are known to respond differently to environmental fluctuations [2–5]. This population heterogeneity leads to loss in process efficiency especially when, due to non-ideal mixing, gradients of process variables arise inside the reactor creating, various local microenvironments [3]. Cells experience these while moving through the reactor, each following an individual path. Additionally to this environmentally induced, extrinsic heterogeneity, further heterogeneity is added by intrinsic perturbations due to the stochasticity of reactions of intracellular molecules that is caused by their low abundance in a cell [3]. Consequently, it is essential to understand single cell physiology to keep population heterogeneity induced yield reduction in industrial scale bioprocesses to a minimum.

Even though population heterogeneity is well-studied as appearing in bioprocesses and especially in response to gradients or different kind of stress [1, 3, 6], mechanistic understanding of this phenomenon is poor. So far, it is only suspected that cells in a bioreactor adapt strategies they also apply in natural habitats to cope with changes in their environment. One of these strategies is noise in gene expression, which is even apparent in a stable environment and was found to affect bioprocess efficiency [6–9]. Thereby, cells diversify their expression profile of certain proteins to have a broader range of response possibilities to environmental changes [7, 10, 11]. Noise influences essential cellular functions like regulation, survival, differentiation and the stress response by a global regulatory feedback system [7, 12–14]. In studies of noise in gene expression an inverse correlation between gene expression and noise could be established [8]. From these findings and the discovery that stress-related genes are noisier than essential genes, the hypothesis was raised that noise in gene expression is used as a regulatory strategy to adjust the level of population heterogeneity depending on whether it is beneficial for the cell population or not [15, 16].

Population heterogeneity in a bioprocess can be conveniently visualized using reporter strains [1, 17]. Reporter strains carry genes for reporter proteins, whose expression can be easily monitored, integrated in their genome, so that they are expressed together with a protein of interest. This enables the collection of snapshots of single cell dynamics following a bioprocess. Different types of reporter strains exist [17, 18]. However, for investigation of population heterogeneity in bioprocesses mostly promoter-based reporter strains are applied. Their fluorescent protein is fused to a promoter responsive to a cellular event of interest. Consequently, fluorescence intensity can be correlated to promoter activity [3, 19]. Possible targets for integration of fluorescent proteins can be confirmed with transcriptomics data, as they allow to predict if a promoter is activated under the conditions that are to be tracked in a bioprocess, e.g. shifts in environmental conditions, exposure to stress of different kind or cellular growth [20–23]. Reporter strains have already been proven to be useful tools in numerous studies of population heterogeneity in lab-scale simulating industrial scale bioprocesses. Examples are investigation of nutrient and oxygen limitation, study of the redox balance or identification of the most robust and best producing subpopulation of cells by integrating a fluorescent protein whose fluorescence output is proportional to product formation [24–27].

Apart from carefully choosing the promoter, the choice of the appropriate fluorescent protein is crucial. A bright spectrum of fluorescence proteins exist and have been employed for generation of reporter strains. The most common is green fluorescent protein (GFP), as its excitation and emission maxima (484/507 nm) fit well and result in high fluorescence intensity in the standard setting of a flow cytometer with a blue laser [28]. But also derivatives of GFP in different colours are applied like blue fluorescent protein (BFP), red fluorescent protein (RFP) and yellow fluorescent protein (YFP) as well as fluorescent proteins of the fruit series, e.g. mStrawberry, mCherry or mPlum (reviewed in [28–30]). When choosing fluorescent proteins, some characteristics should be considered like its maturation time as it is, together with its photostability, crucial when fast changes are to be monitored. For instance, while wildtype GFP has a maturation time of around 60 min in *E. coli*, the maturation time of EmeraldGFP (EmGFP), a modified version thereof, is just 12 min [31]. Furthermore, also a possible dependence of the fluorescence signal on environmental factors, like temperature, oxygen and pH has to be considered.

When investigating population heterogeneity in bioprocesses with reporter strains, the resulting fluorescence distributions are most often visualized with flow cytometry (for reviews see [32, 33]). This high-throughput technique enables visualization of different subpopulations in a reactor, while at the same time also collecting information about single cells in a sample. As the instruments gradually advance, it is possible to simultaneously measure more and more fluorescence parameters with high resolution [3]. Hence, fluorescent proteins have to also be fitted to not interfere with each other, exhibit spectral interaction or distributions that are superimposed. Additionally, their fluorescence characteristics (excitation/emission) have to be compatible with the flow cytometers’ lasers and filters [1]. However, so far mostly single reporter strains, expressing one fluorescent protein to follow one cellular event of interest, are used when studying population heterogeneity [3, 12]. Then, other cellular characteristics remain in the dark, if one does not deploy global omics-methods. Nevertheless, even these are only partly available with single cell resolution or possible with big invest in labour and time [34, 35].

Our goal was to construct and characterize triple reporter strains based on one of the most important industrial production hosts *E. coli* BL21(DE3) and a modified version thereof, *E. coli* T7E2, which enable to follow three essential cellular characteristics simultaneously to raise the information content gained from studies of population heterogeneity in bioprocesses. To track growth rate of single cells, the strains express EmGFP together with the ribosomal promoter *rrnB*. Furthermore, mStrawberry is integrated downstream of the alternative sigma factor 38, whose expression is correlated with the general stress response of single cells. The third marker is integrated to follow oxygen limitation of single cells. For that purpose, TagRFP657, a variant of RFP, is placed in the *nar*GHIJ gene cluster. Since the expression of these three characteristics are interlinked, the triple reporter strains should also enable to observe correlations between the different markers. All markers have been previously employed as single reporters of population heterogeneity in bioprocesses, but never combined in one experimental setup that enables the direct correlation of the three cellular characteristics on single cell level [22, 36, 37]. This is to our knowledge the first time to employ a triple reporter strain to investigate population heterogeneity in bioprocesses. The only other approach we are aware of, was built to study promoter activities related to quorum sensing, biosynthesis of galactoglucan and viability with three different fluorescent proteins [38].

In *E. coli* the number of ribosomes is proportional to the growth rate to fulfil the requirements for protein synthesis [39]. The ribosomal promoter *rrnB* is part of one of two operons compromising in total seven copies of *rrn* in the cell. Since the *rrnCABE*-operon is located in close vicinity to the origin of replication on the chromosome, its number is significantly increased in fast-growing cells compared to slow-growing cells. Thus, the rate of rRNA synthesis will be higher in fast-growing cells due to their increased gene dosage. The promoter activity can therefore be correlated to single cell growth which was also shown in transcriptomics studies and studies of cells from batch and chemostat cultures [19, 36, 40].

Expression of the alternative sigmafactor 38 *rpoS* is induced in response to various kind of stress, e.g. starvation and nutrient limitation and influences the expression of around 500 genes to enable the cell to cope with the stress experienced. Thereby it has, among others, influence on intracellular pH regulation, the metabolism and its flexibility and the growth rate [41]. Furthermore, *rpoS* expression plays an important role when cells change from exponential growth to stationary phase, which is accompanied by a steep increase in *rpoS* levels [22]. Earlier, *rpoS* reporter strains could reveal differences between the general stress response of cells in well-mixed fed-batch processes and the equivalent scale-down reactor [12].

The *nar*GHIJ operon was found to be upregulated upon dissolved oxygen depletion [8]. It codes for the alpha subunit of a nitrate reductase, which allows *E. coli*, in conjugation with NADH-dehydrogenase, to use nitrate as an electron acceptor during anoxic growth while still gaining energy through oxidative phosphorylation [42, 43]. In cultivation studies its expression was found to be upregulated when oxygen saturation in the extracellular environment drops below 40% [37].

*Escherichia coli* BL21(DE3) has several advantageous characteristics like low acetate production at high glucose levels as well as being protease deficient [44]. The second strain used in this study, *E. coli* T7E2, should be more robust than its ancestor in an industrial cultivation setup [45]. Its modifications are related to removal of major parts of the lambda DE3 prophage and replacement of the cryptic prophage Rac by the *pgl* gene encoding 6-phosphogluconolactonase which BL21(DE3) has lost and that prevents accumulation of sugar-intermediates.

## Methods

### Triple reporter strains

Two *Escherichia coli* triple reporter strains, *E. coli* G7_BL21(DE3)_ and *E. coli* G5_T7E2_, expressing three chromosomally integrated fluorescent proteins connected to the expression of three different cellular markers (see below and Additional file 1: Table A1) were characterized in this study. *E. coli* G7_BL21(DE3)_ originates from wildtype *E. coli* BL21(DE3), whereas *E. coli* G5_T7E2_ is based on a modified version of *E. coli* BL21(DE3), T7E2 [45]. Additionally, during initial characterization experiments the corresponding single- and double reporter strains were employed (see Additional file 1). All strains were synthesized by Gene Bridges (Heidelberg, Germany) and stored as 15% glycerol stocks at − 80 °C. Wildtypes *E. coli* BL21(DE3) and *E. coli* T7E2 were used as experimental reference.

#### Genetic modifications

Wildtype *E. coli* BL21(DE3) as well as *E. coli* T7E2 were altered by a series of *knock*-*in* recombinations with synthetic cassettes. Each reporter protein was thereby under transcription control of a certain native promoter. The mStrawberry gene was inserted downstream of *rpoS* in conjunction with its own ribosomal binding site (RBS) without removing any native sequence. The second reporter TagRFP657 is placed behind the *nar*GHIJ gene cluster without removing any native sequence. For the third marker a synthetic copy of the *rrnB* promoter complex together with EmGFP(A206K) and a consensus RBS in front and a transcriptional terminator behind the open reading frame (ORF) was integrated in the rhamnose operon. The *knock*-*in* was performed removing 833 bp of native sequence (5′-terminal 293 bp of *rhaS* to 5′-terminal 453 bp of *rhaB*). In all of these transcriptional fusions a strong synthetic RBS (5′-AAAGAGGAGAAA-3′) [46] was used, followed by six intervening nucleotides until the start of the respective ORF, to ensure efficient translation of the fluorescence protein. Each of the three DNA cassettes was integrated into the bacterial genome in connection with bilaterally recombinase flippase (FLP) recognition target sequence [47] flanked antibiotic resistance markers. This allowed for subsequent removal of antibiotic markers by FLP recombination steps, so that none of the strains carries an antibiotic resistance. Correct insertion and orientation were confirmed by amplification of the corresponding loci using locus specific primer via PCR and subsequent Sanger sequencing of the obtained amplicon (data not shown).

### Bioreactor cultures

#### Pre-cultures

Single colonies from minimal medium [48] agar plates of the triple-reporter strains and the respective wildtypes were used to inoculate 50 mL minimal medium [48] cultures in 500 mL baffled shake flasks. The flasks were shaken for around 6 h at 150 rpm and 37 °C. The minimal medium was composed of 13.3 g L^−1^ KH_2_PO_4_, 4 g L^−1^ (NH_4_)_2_HPO_4_, 1.859 g L^−1^ citric acid ·H_2_O, 20 g L^−1^ glucose, 1.2 g L^−1^ MgSO_4_·7H_2_O and 1 mL^−1^ of a trace metal solution (0.125 g L^−1^ CoCl_2_·6H_2_O, 0.75 g L^−1^ MnCl_2_·4H_2_O, 0.075 g L^−1^ CuCl_2_·2H_2_O, 0.15 g L^−1^ H_3_BO_3_, 0.125 g L^−1^ Na_2_MoO_4_·2H_2_O, 0.42 g L^−1^ EDTA, 0.4 g L^−1^ Zn(CH_3_COO)_2_*2H_2_O and 3 g L^−1^ Fe(III)citrate). To avoid foaming the medium was supplemented with 1 mL^−1^ Antifoam 204 (Sigma Aldrich, MO, USA). Stirred-tank bioreactors were inoculated with the volume necessary to achieve an optical density at 600 nm (OD_600_) of 0.02.

#### Batch processes

The stirred-tank bioreactor employed in this study was a 1.5 L reactor (Labfors 5, Infors AG, Switzerland) with a working volume of 1.2 L. Cells were cultured in minimal medium according to Riesenberg et al. [48] with an initial glucose concentration of 20 g L^−1^. Sensors for pH and dissolved oxygen (EasyFerm Bio HB and VisiFerm DO ECS, respectively, Hamilton Bonaduz AG, Switzerland) were calibrated according to standard procedures using a two- and one-point calibration, respectively (pH 4.02 and pH 9.21, calibration for 100% dissolved oxygen). The pH was controlled at pH 6.8 with 1 M ammonia. Temperature and stirring were kept constant at 37 °C and 1000 rpm. Aeration was 2 vvm in the beginning and automatically increased in 0.5 steps until 8 vvm to avoid oxygen limitation. Continuous analysis of off-gas carbon dioxide (CO_2_) was performed using a gas sensor (BlueInOne Ferm, BlueSens, Germany). Samples for OD_600_, high performance liquid chromatography (HPLC), dry cell weight (DW) measurements and flow cytometry (FC) analysis were withdrawn frequently following all phases of a typical batch culture of *E. coli*. Samples for OD_600_, FC analysis and DW were analyzed directly. Samples for HPLC analysis were centrifuged for 5 min at 16.438 g, filtrated (pore size 0.2 µm) and kept at − 20 °C until analysis. All experiments were performed in triplicates and variation is considered using error bars.

#### Continuous processes

Aerobic, glucose-limited, level-based chemostat cultures were started after an initial batch phase performed as described above. After glucose depletion, detected by a rapid rise in dissolved oxygen in the reactor, cultures were switched to chemostat operation. A constant feed with the same composition as the batch medium was applied to reach respective dilution rates. Steady state was confirmed by constant values for O_2_, exhaust CO_2_ and DW after five mean hydraulic residence times. When steady state was verified, cultures were either sampled at different dilution rates or perturbed with additional substrate and oxygen limitation.

#### Change in dilution rate

Chemostat cultures with the two triple reporter strains were run with different dilution rates. For *E. coli* G7_BL21(DE3)_, chemostats were run at dilution rates of 0.1 h^−1^, 0.2 h^−1^, 0.25 h^−1^, 0.3 h^−1^ and 0.4 h^−1^. For *E. coli* G5_T7E2_, chemostats were run at dilution rates of 0.14 h^−1^, 0.2 h^−1^, 0.3 h^−1^ and 0.4 h^−1^. After verification of steady state, three samples for OD_600_, DW, HPLC and FC analysis were taken during three successive residence times. Samples were taken in triplicates and treated as described for batch cultures. Variation between replicates is considered using error bars.

#### Pulses of glucose and oxygen limitation

Chemostat cultures were run with a dilution rate of 0.2 h^−1^. After verification of steady state, cultures were simultaneously perturbed with a final concentration of 2 g L^−1^ additional glucose in the reactor and reduced oxygen availability. For this purpose, aeration was turned off for 30 min. The perturbation was followed by frequent sampling before (60 min and 30 min prior to perturbation), during shutdown of aeration (0 min, 5 min, 10 min and 20 min) and after perturbation (40 min, 60 min, 90 min and 120 min) for OD_600_, DW, HPLC and FC analysis. Samples were taken in triplicates and treated as described above. Variation between replicates is considered using error bars.

### Sample analysis

#### Optical density and dry cell weight

An UV–Vis spectrophotometer (Genesys 10uv-vis, Thermo Scientific, MA, USA) was used for at-line measurement of OD_600_. For biomass determination (DW), 2 mL cell suspension was centrifuged for 5 min at 16.438 g in pre-dried (48 h at 80 °C) and weighted Eppendorf tubes. The pellet was dried for 48 h at 80 °C, cooled down to room temperature (RT) and weighted. DW was determined by weight-difference between empty and filled tube. After measurement of OD_600_ and DW in batch cultures, a correlation was established through linear regression (Additional file 2).

#### Flow cytometry analysis

Samples taken from the reactor were diluted in deionized water adjusted to the dilution used for OD_600_ measurements. Diluted samples were centrifuged for 1 min at 16.438 g. The pellet was re-suspended in 1 mL sterile 0.9% saline (NaCl) solution by vortexing. Afterwards cells were kept at RT until analysis of EmGFP, mStrawberry and TagRFP657 fluorescence. For FC analysis a CytoFLEX (Beckman Coulter, CA, USA) equipped with two lasers 488 nm and 638 nm was used. The minimum laser power was 50 mW and the beam spot size 5 µm × 80 µm. The FC has nine detecting channels for FSC, SSC and seven fixed wavelengths (525/40 nm, 585/42 nm, 610/20 nm, 660/20 nm and 2× 780/60 nm) avoiding spectral overlap. To eliminate background noise, the detection thresholds were set in SSC. 100,000 events were recorded with a rate of ∼ 1000 events/s (around 20 µL/s). During measurement, fluorescence distributions were visualized with the FC proprietary software CytExpert. For all experiments, measured fluorescence was put in relation to respective negative controls (see Additional file 1). All obtained results were saved in FCS 3.0 format.

#### HPLC analysis

Concentrations of glucose, succinate, acetate, citrate, malate, lactate and formate were quantified by HPLC (Finnigan Surveyor, Thermo Fisher Scientific, Waltham, USA). Detection was performed by refractive index (RI) (RID Agilent 1200, Agilent Technologies, CA, USA) with a 300 mm × 7.8 mm Aminex HPX-87H ion exchange column (Bio-Rad, Hercules, CA, USA). 20 µL sample was injected into the mobile phase of 5 mM H_2_SO_4_ (aq.), temperature was 60 °C and flow rate 0.35 mL min^− 1^. The results from HPLC analysis were used together with DW measurements to calculate yield coefficients. Triplicate datasets are considered using error bars.

### Data analysis

Data analysis was performed using MATLAB^®^ R2017a (The MathWorks, Inc., Natick, MA, USA). Flow cytometry files were loaded using the function fcsread and saved into mat files (by L. Balkay, University of Debrecen, Hungary, available on MATLAB^®^ central file sharing). Applying the MATLAB^®^ built-in hist function to the recording channels of the FC, cell count in each channel was saved and enabled histogram plots with relative cell count vs. fluorescence intensity for each detector. To account for variance in fluorescence distributions, which correlates for fluorescence originating from cellular characteristics with noise in gene expression, the coefficient of variance (CV) was determined. The CV is defined as the ratio between standard deviation and mean of a distribution, which were calculated using the MATLAB^®^ built-in functions mean and std. Consequently, plots for CV against mean fluorescence respectively plots for CV and mean fluorescence intensity were generated for all fluorescent proteins. Normalized mean EmGFP fluorescence was estimated dividing mean EmGFP fluorescence by OD_600_. Linear regression analysis was performed employing the MATLAB^®^ built-in polyfit function with a degree of one. The result was evaluated using the MATLAB^®^ built-in polyval function.

## Results

### Batch cultivation of triple reporter strains in comparison to the wildtype

After preliminary characterization in shake flask cultures and confirmation that all fluorescence proteins are inducible under relevant bioprocess conditions and the expression of the fluorescent proteins is not a burden to the cells respectively their fluorescence is pH insensitive (see Additional file 1), biological triplicate batch cultivations in stirred-tank bioreactors with minimal medium were performed for the triple reporter strains *E. coli* G5_T7E2_ and *E. coli* G7_BL21(DE3)_ as well as for the wildtypes *E. coli* BL21(DE3) and *E. coli* T7E2. Population level growth was followed by OD_600_ and biomass concentration. Furthermore, consumed respectively produced metabolites including the off-gas composition were followed. On single cell level, growth (expression of *rrnB*-EmGFP), general stress response (expression of *rpoS*-mStrawberry) and oxygen limitation (expression of *nar*-TagRFP657) of the triple reporter strains *E. coli* G5_T7E2_ and *E. coli* G7_BL21(DE3)_ were measured with flow cytometry.

### Population level physiology

**Fig. 1:**
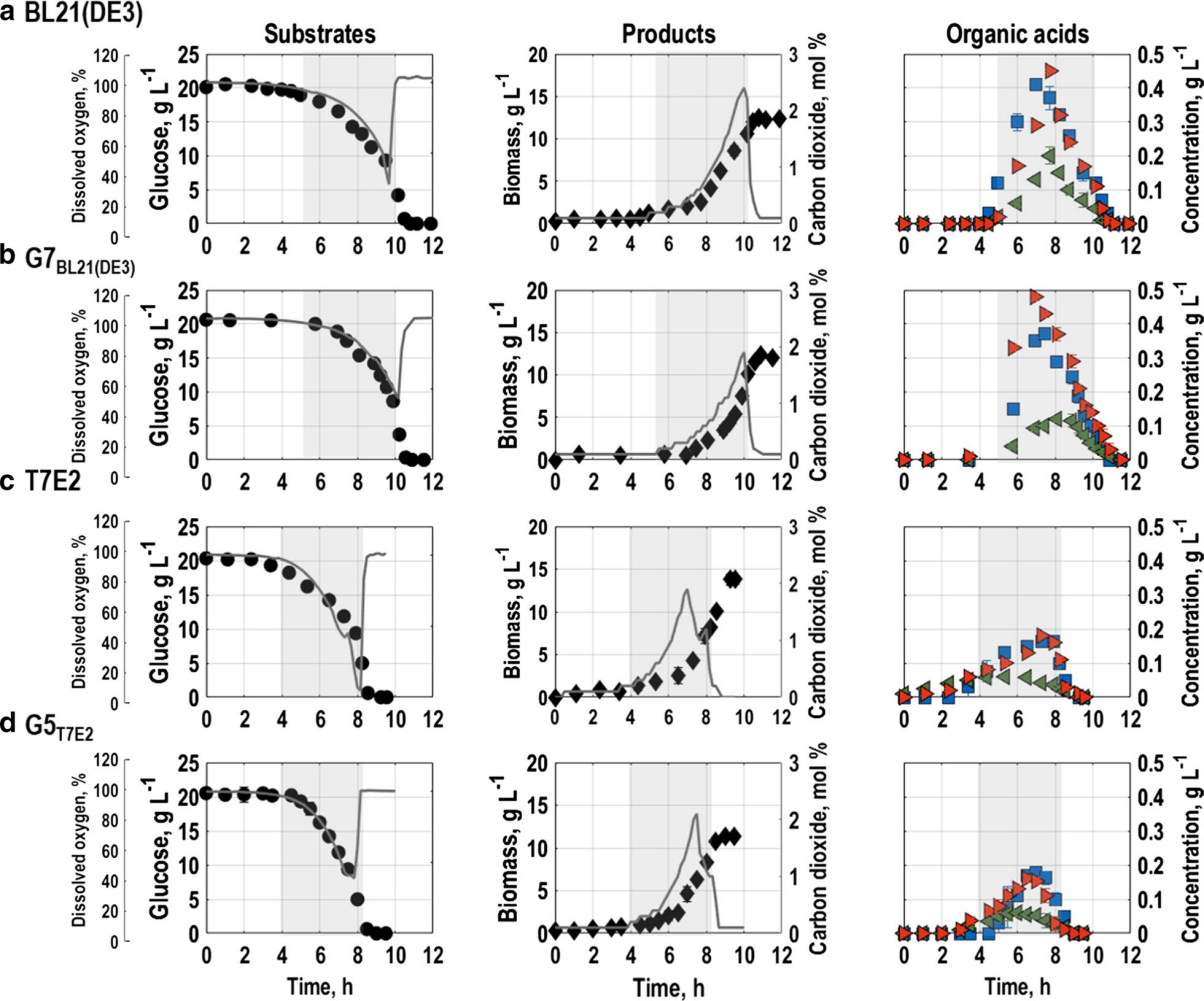
Biomass, metabolites (glucose, acetate (blues squares), malate (green left-pointing triangle) and formate (red right-pointing triangle)) concentrations, off-gas carbon dioxide and dissolved oxygen following batch cultures in stirred-tank bioreactors with the triple reporter strains *E. coli* G7_BL21(DE3)_ (**b**) and *E. coli* G5_T7E2_ (**c**) in comparison to wildtypes *E. coli* BL21(DE3) (**a**) and *E. coli* T7E2 (**d**) with minimal medium (T = 37 °C, pH = 6.8, initial glucose concentration 20 g L^−1^). Error bars indicate triplicate datasets. Grey areas mark the exponential growth phases of the different strains

Generally, the wildtypes *E. coli* BL21(DE3) and *E. coli* T7E2 as well as the triple reporter strains, *E. coli* G7_BL21(DE3)_ and *E. coli* G5_T7E2_, showed a typical growth behaviour. After inoculation, a lag phase of around five hours for the wildtype *E. coli* BL21(DE3) and *E. coli* G7_BL21(DE3)_ was characterized by constant values for glucose concentration and dissolved oxygen as well as no significant rise in carbon dioxide or biomass concentration (Fig. 1a, b, substrates and products). Furthermore, no production of organic acids could be detected (Fig. 1a, b, organic acids). Also *E. coli* T7E2 and *E. coli* G5_T7E2_ exhibited a lag-phase, which however was around 1 h shorter than for the other strains (Fig. 1c, d, substrates and products). Following the lag phase, the cells grew exponentially consuming the glucose in the medium and producing carbon dioxide, biomass, acetate and small amounts of malate and formate (see Fig. 1a–d, exponential phase indicated as grey area). While the wildtype *E. coli* BL21(DE3) and the triple reporter strain *E. coli* G7_BL21(DE3)_ did not produce any further metabolites, *E. coli* T7E2 and *E. coli* G5_T7E2_ additionally produced minor amounts of citrate, lactate and succinate (total 2% respectively 2.5% of overall carbon, data not shown). The maximum specific growth rate was similar for all strains (µ_G5 T7E2_ = 0.728 ± 0.008 h^−1^; µ_T7E2_ = 0.727 ± 0.005 h^−1^; µ_G7 BL21(DE3)_ = 0.719 ± 0.007 h^−1^; µ_BL21(DE3)_ = 0.723 ± 0.004 h^−1^) and in accordance with earlier studies for *E. coli* BL21(DE3) [49, 50].

From mid-exponential growth phase onwards, *E. coli* G7_BL21(DE3)_ and its wildtype re-assimilated the prior produced acetate, malate and formate simultaneously with consumption of residual glucose (Fig. 1a, b, organic acids). In contrast, *E. coli* G5_T7E2_ and its wildtype started consuming secondary carbon sources first towards the end of the exponential growth phase (Fig. 1c, d, organic acids). This short second growth phase is visible as small second rise in carbon dioxide. Afterwards, depletion of all metabolites in the cultures is characterized by a steep rise in air saturation in the reactor accompanied by a decrease in carbon dioxide levels for all cultured strains (Fig. 1a–d, substrate and products). Consequently, the cells reach stationary phase, where no further increase in biomass concentration is found (Fig. 1a–d, products). All strains reached a final biomass concentration of around 12 g L^−1^ at the end of the exponential growth phase, which is comparable to published data for *E. coli* BL21(DE3) [51, 52].

Due to the shorter lag-phase, the batch processes with *E. coli* T7E2 and *E. coli* G5_T7E2_ are slightly shorter than for the two other strains. Moreover, *E. coli* G5_T7E2_ and its wildtype produced lower amounts of acetate compared to *E. coli* G7_BL21(DE3)_ and the wildtype *E. coli* BL21(DE3) (Table 1). Apart from that, there is no significant difference comparing yield coefficients for the four strains and carbon balances closed with at least 98%.

**Table 1:** Carbon balances and yield coefficients for biomass (X), CO_2_, acetate (Ac), formate (For) and malate (Mal) with the substrate glucose (S) of batch cultivations in stirred-tank bioreactors of the two triple reporter strains *E. coli* G7_BL21(DE3)_ and *E. coli* G5_T7E2_ in comparison to wildtypes *E. coli* BL21(DE3) and *E. coli* T7E2 with minimal medium

Strain	BL21(DE3)	G7_BL21(DE3)_	T7E2	G5_T7E2_
Yield, Cmole Cmole^−1^				
Y_XS_	0.64 ± 0.03	0.68 ± 0.03	0.68 ± 0.04	0.69 ± 0.02
Y_CO2S_	0.19 ± 0.02	0.16 ± 0.01	0.18 ± 0.02	0.19 ± 0.02
Y_AcS_	0.08 ± 0.01	0.08 ± 0.02	0.03 ± 0.01	0.03 ± 0.03
Y_ForS_	0.06 ± 0.02	0.04 ± 0.02	0.07 ± 0.03	0.07 ± 0.02
Y_MalS_	0.04 ± 0.02	0.02 ± 0.02	0.03 ± 0.01	0.02 ± 0.01
Carbon balance	1.01 ± 0.03	0.98 ± 0.03	0.99 ± 0.03	0.99 ± 0.02

*Y*_*XS*_ biomass yield with glucose, *Y*_*CO2S*_ yield of CO_2_ with glucose, *Y*_*AcS*_ yield of acetate with glucose

All values are given as mean with error bars originating from biological triplicates

Expectedly, comparing *E. coli* G7_BL21(DE3)_ and its wildtype, the strains exhibit strong resemblance in their growth behaviour in batch cultures, which further confirms, that expression of the fluorescent proteins is not a burden to the cells. *E. coli* G5_T7E2,_ and its wildtype however, behave similar but slightly different from the other two strains (see also Additional file 2). One reason could be, that *E. coli* T7E2 carries a knock-in of *pgl* that encodes for 6-phosphogluconolactonase and which *E. coli* BL21(DE3) lacks [45]. A loss of this gene leads to a blockage of the pentose-phosphate-pathway, so that intermediates of the remaining part of the central carbon metabolism can be stronger accumulated.

### Single cell level physiology

#### Single cell growth

**Fig. 2:**
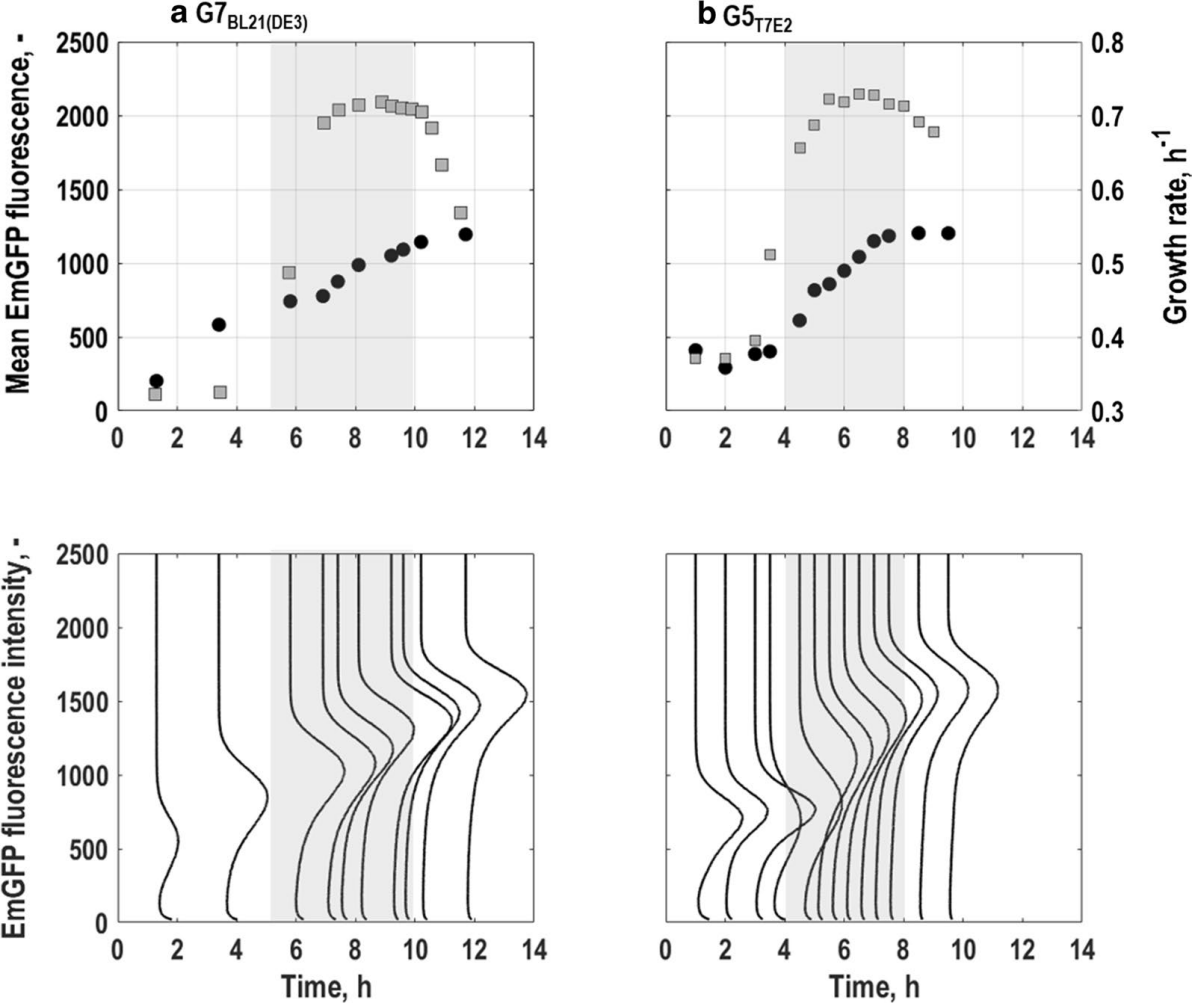
Single cell level physiology for the triple reporter strains *E. coli* G7_BL21(DE3)_ (**a**) and *E. coli* G5 _T7E2_ (**b**) following batch cultures in stirred-tank bioreactors with minimal medium (T = 37 °C, pH = 6.8, initial glucose concentration 20 g L^−1^). Fluorescence distributions (lower subplots) and mean fluorescence characteristics (upper subplots) for single cell growth related to *rrnB*-EmGFP expression are depicted together with the course of population level growth rate. All values are shown as average of triplicate measurements following the batch. Grey areas mark the exponential growth phases of the different strains

The growth of single cells could be followed by expression of EmGFP together with the ribosomal promoter *rrnB* (Fig. 2). During batch cultivations, in agreement with the course of biomass concentration (Fig. 1b, products) and the growth rate on population level (Fig. 2a), EmGFP-expression of *E. coli* G7_BL21(DE3)_ remained low during the first 5 h (Fig. 2a). Thereafter, *E. coli* G7_BL21(DE3)_ started to grow exponentially (Fig. 1, grey area), which led to a steep increase in growth rate on population level and a rise in fluorescence intensity of EmGFP (Fig. 2a). Then, during exponential growth, the growth rate on population level remained about constant, while EmGFP-expression and thereby its mean constantly increased further (Fig. 2a, grey area). With glucose depletion after around 10 h of batch culture (Fig. 1b, substrates), EmGFP-expression intensity endured at constant values until the end of the batch, while the growth rate on population level decreased (Fig. 2a).

EmGFP-expression of *E. coli* G5_T7E2_ showed a similar trend as for *E. coli* G7_BL21(DE3)_, however the different growth phases of the batch culture were clearer reflected by EmGFP-expression of *E. coli* G5_T7E2_ (Fig. 2a, b). Consistent with the shorter lag-phase on population level (Fig. 1d, substrates and products), the growth rate on population level of *E. coli* G5_T7E2_ as well as the expression of EmGFP started to rise earlier than for *E. coli* G7_BL21(DE3)_, after around 4 h of cultivation (Fig. 2b). During exponential growth (Fig. 2b, grey area), EmGFP-expression and thereby its mean increased further until the beginning of stationary phase after around 8 h of batch cultivation, where it stagnated (Fig. 2b). As expected and similar to *E. coli* G7_BL21(DE3)_, the population level growth rate remained about constant during exponential growth and slowly decreased with glucose depletion (Fig. 2b).

Generally, no subpopulation division was found in any phase of the batch culture, which is consistent with earlier studies [53–55]. However, the fluorescence distributions varied in shape and fluorescence intensity. These changes can be described by the coefficient of variance (CV) of a distribution that represents the ratio between its standard deviation and its mean fluorescence intensity and can also be correlated to noise in gene expression [56]. The CV of EmGFP fluorescence distributions of *E. coli* G7_BL21(DE3)_ (Fig. 5a) was high during lag-phase, but decreased with increasing mean fluorescence until the beginning of the exponential growth phase (compare with Fig. 1b, substrates). During exponential growth, mean fluorescence intensity further increased while the CV only gradually decreased until the end of the process. This is in agreement with studies that revealed low noise levels for stronger expressed genes whereas the opposite is observed when expression levels are low [15, 57]. Compared to *E. coli* G7_BL21(DE3)_, *E. coli* G5_T7E2_ exhibited a similar mean fluorescence intensity and CV at the end of the process (Fig. 5a). Strikingly, fluorescence distributions broadened right before the onset of exponential growth phase, then, during exponential growth their CV decreased significantly, which was also found earlier [15, 58]. Also for *E. coli* G5_T7E2_ the CV revealed an inverse correlation between noise in EmGFP-expression and EmGFP-expression (Fig. 5a).

#### General stress response of single cells

**Fig. 3:**
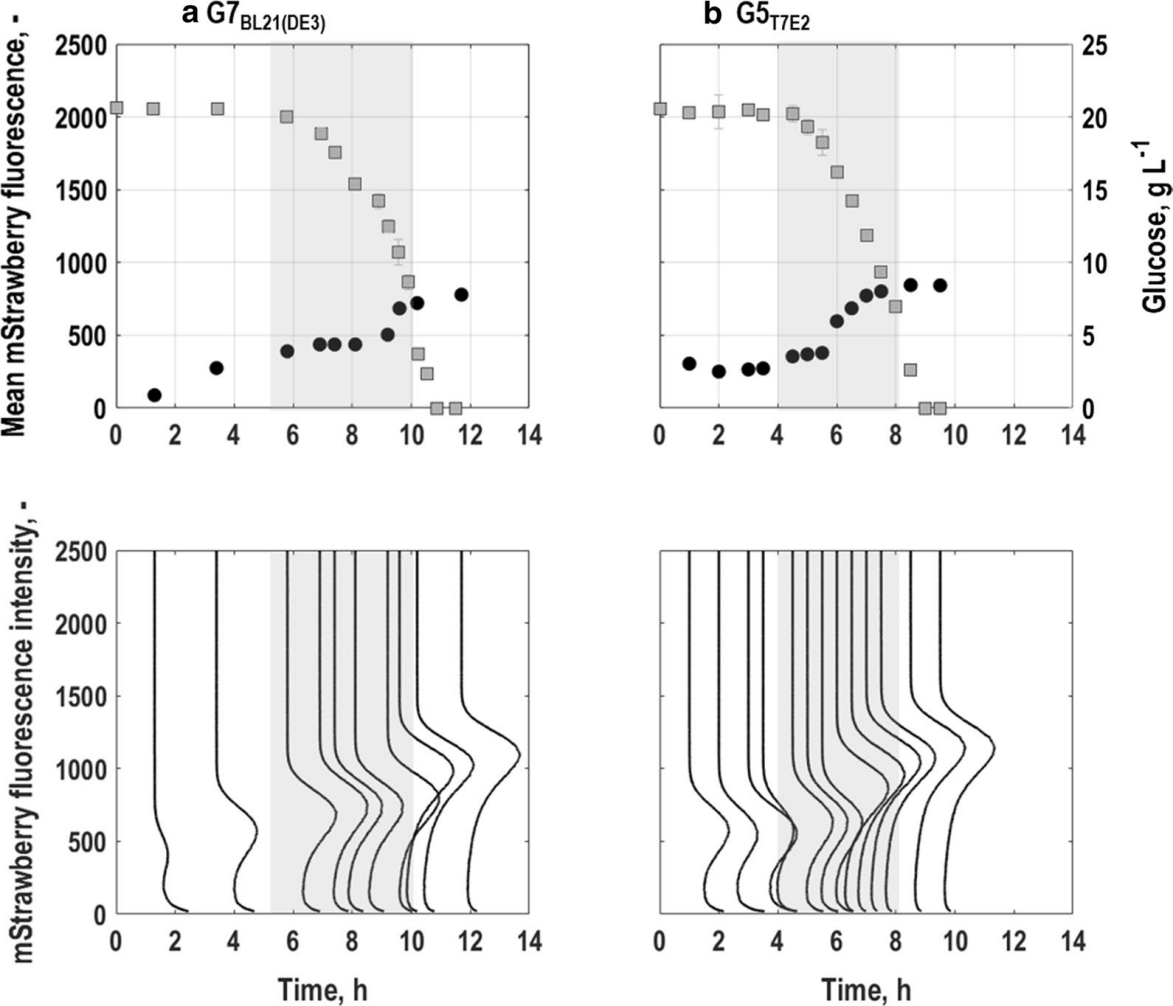
Single cell level physiology for the triple reporter strains *E. coli* G7_BL21(DE3)_ (**a**) and *E. coli* G5 _T7E2_ (**b**) following batch cultures in stirred-tank bioreactors with minimal medium (T = 37 °C, pH = 6.8, initial glucose concentration 20 g L^−1^). Fluorescence distributions (lower subplots) and mean fluorescence characteristics (upper subplots) for the general stress response of single cells related to *rpoS*-mStrawberry expression is depicted together with substrate availability. All values are shown as average of triplicate measurements following the batch. Grey areas mark the exponential growth phases of the different strains

General stress response of single cells was tracked by the expression of *rpoS* in combination with mStrawberry (Fig. 3). For *E. coli* G7_BL21(DE3)_, irrespective of a moderate upshift at the beginning of the lag-phase (compare with Fig. 1b), expression of mStrawberry remained low during glucose consumption until mid-exponential growth phase (around 9 h of cultivation, grey area, Fig. 3a). Then, when the glucose concentration fell below 5 g L^−1^ and during transition to stationary phase, mStrawberry-expression rose until the end of the batch process and depletion of the glucose in the reactor (Fig. 3a).

Again, *E. coli* G5_T7E2_ showed a similar trend in mStrawberry-expression than *E. coli* G7_BL21(DE3)_. At the beginning of the process, expression levels were low and remained about constant until mid-exponential growth phase (compare with Figs. 1d, 3b, grey area). Then, mStrawberry-expression significantly increased until the end of the process and depletion of the glucose in the reactor (Fig. 3b). Compared to *E. coli* G7_BL21(DE3)_, mean fluorescence intensity reached slightly higher values at the end of the cultivation and additionally the rise in fluorescence intensity around mid-exponential growth phase was detected earlier for *E. coli* G5_T7E2_ than for *E. coli* G7_BL21(DE3)_.

As for EmGFP-expression, the development of the CV was for both strains, *E. coli* G5_T7E2_ and for *E. coli* G7_BL21(DE3)_, inversely correlated to that of mean fluorescence intensity with highest levels of noise in mStrawberry-expression at the beginning of the process (Fig. 5b). Then, the CV decreased until mid-exponential phase and thereafter further, however with a lower rate (Fig. 5b).

#### Oxygen limitation of single cells

**Fig. 4:**
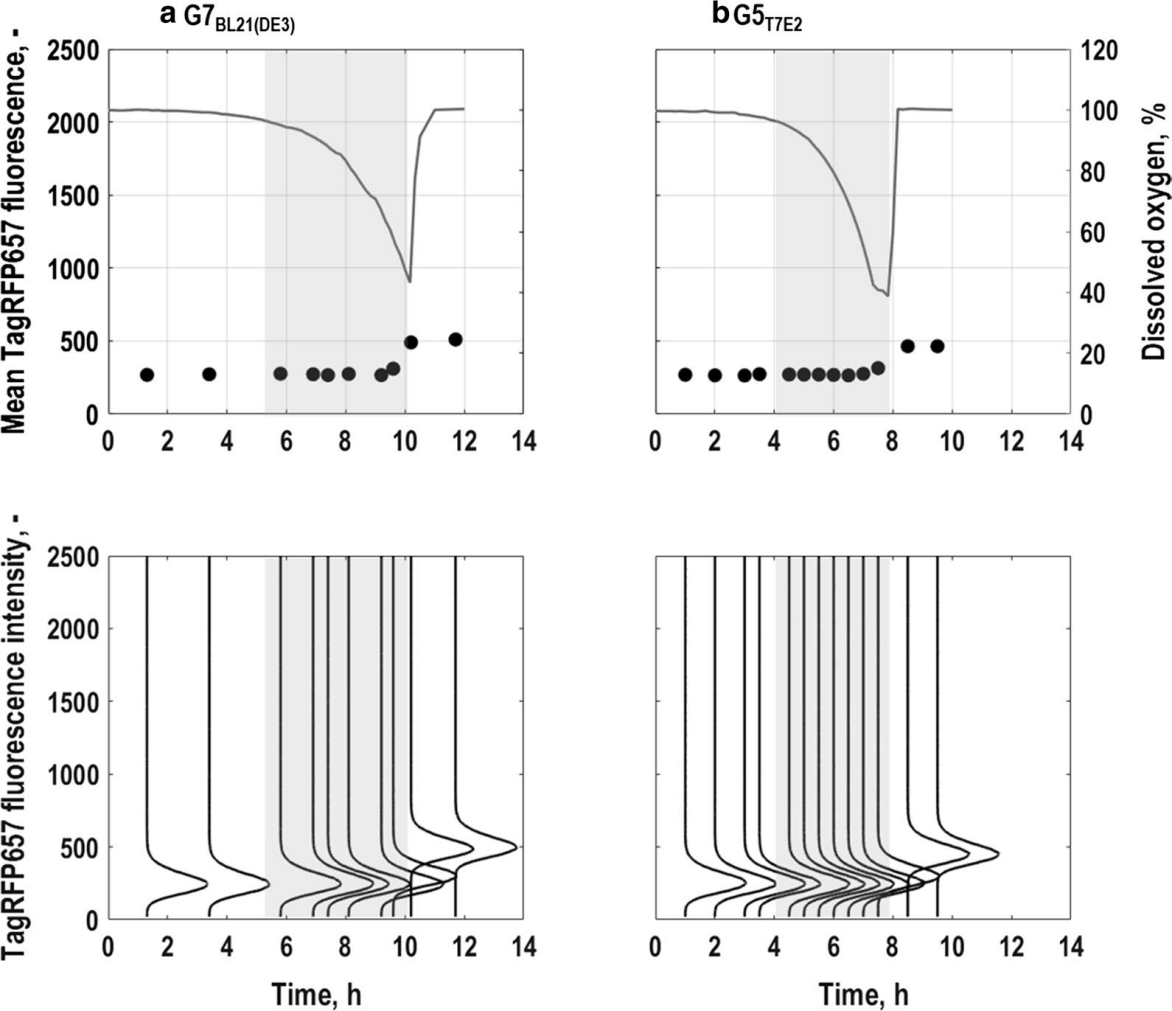
Single cell level physiology for the triple reporter strains *E. coli* G7_BL21(DE3)_ (**a**) and *E. coli* G5 _T7E2_ (**b**) following batch cultures in stirred-tank bioreactors with minimal medium (T = 37 °C, pH = 6.8, initial glucose concentration 20 g L^−1^). Fluorescence distributions (lower subplots) and mean fluorescence characteristics (upper subplots) of oxygen limitation of single cells which is followed by *nar*-TagRFP657 expression, is correlated to dissolved oxygen in the bioreactor. All values are shown as average of triplicate measurements following the batch. Grey areas mark the exponential growth phases of the different strains

**Fig. 5:**
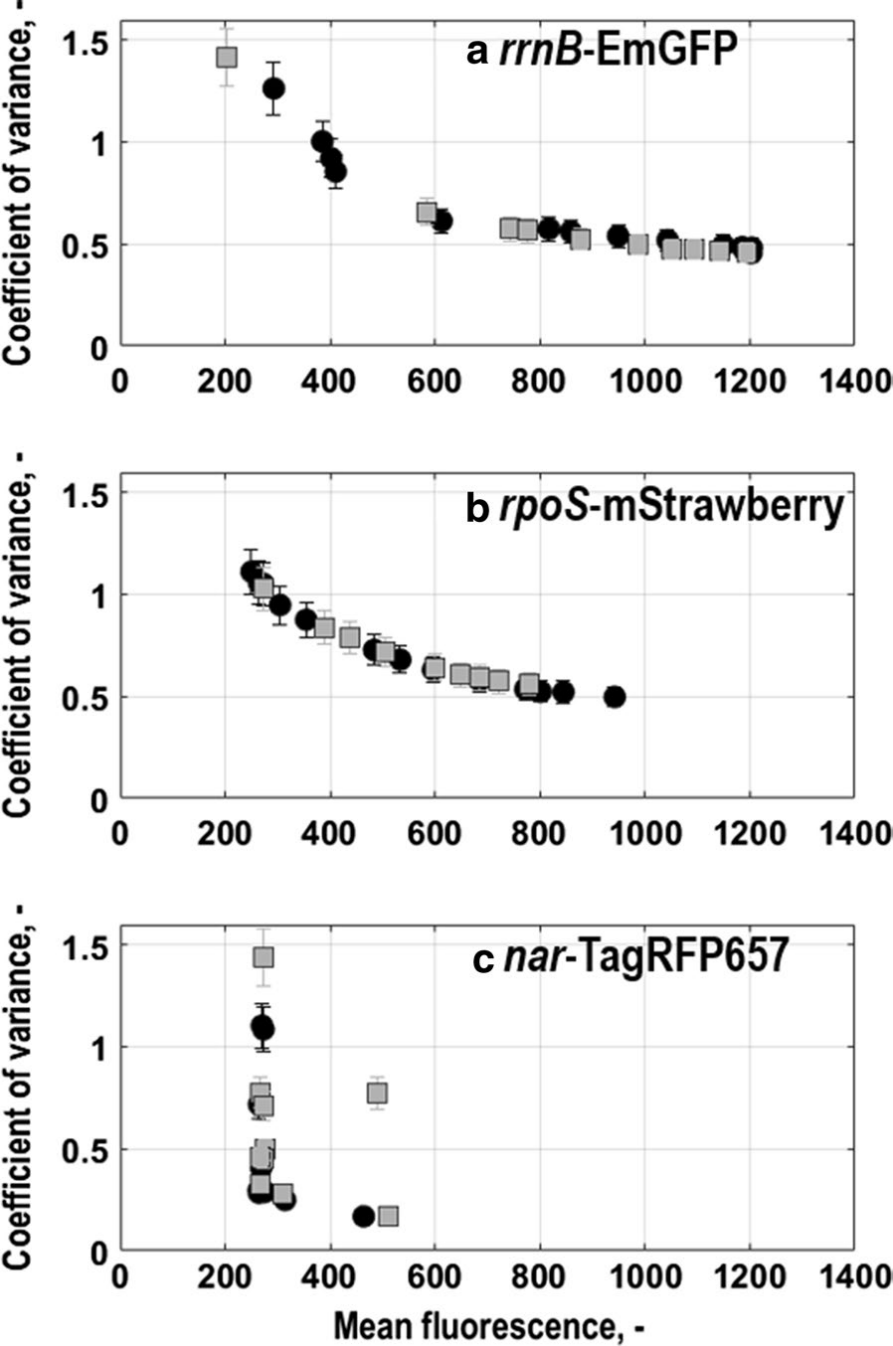
Coefficient of variance vs. mean fluorescence intensity for the triple reporter strains *E. coli* G7_BL21(DE3)_ (grey squares) and *E. coli* G5_T7E2_ (black circles) grown with minimal medium in batch cultures in stirred-tank bioreactors (T = 37 °C, pH = 6.8, initial glucose concentration 20 g L^−1^). Values for single cell growth related to *rrnB*-EmGFP expression (**a**), general stress response of single cells related to *rpoS*-mStrawberry expression (**b**) and oxygen limitation of single cells related to *nar*-TagRFP657 expression (**c**) are depicted as average of triplicate measurements

The expression of the *nar*-operon in combination with TagRFP657 was used to track oxygen limitation of single cells (Fig. 4). Generally, expression levels of this marker were lower than for the other markers, nevertheless a distinct fluorescence upshift under oxygen limited conditions could be detected. For both triple reporter strains, *E. coli* G7_BL21(DE3)_ and *E. coli* G5_T7E2_, TagRFP657-expression only slightly rose during the transition from exponential growth phase to stationary phase where oxygen saturation in the reactor dropped to close to 40% (Fig. 4a, b). Mean expression levels were similar for *E. coli* G7_BL21(DE3)_ and *E. coli* G5_T7E2_. Interestingly, TagRFP657-expression was found to be induced earlier than in previous studies where *nar*- expression first rose when oxygen saturation in the reactor fell below 40% [37]. Afterwards, expression levels remained elevated until the end of the cultivation (Fig. 4a, b), probably related to a higher half-life of TagRFP657, while the air saturation returned to 100% with the depletion of the substrate in the reactor (Fig. 1b, d). Considering the correlation between mean fluorescence intensity and CV (Fig. 5c), as for the other markers, noise in gene expression seem to decrease with increasing mean fluorescence intensity, but the trend is less clear than for expression of the other fluorescence markers. Moreover, the CV seemed to fluctuate for *E. coli* G7_BL21(DE3)_, especially towards the end of the process (Fig. 5c).

### Population heterogeneity in chemostat cultures with the triple reporter strains

Since in batch cultures, all fluorescent proteins could be detected and showed the fluorescence pattern throughout different process phases that were expected due to their characteristics, the triple reporter strains *E. coli* G7_BL21(DE3)_ and *E. coli* G5_T7E2_ were applied for investigation of population heterogeneity in continuous cultures in two case studies. As for batch cultures, all experiments were followed with frequent sampling for population and single cell level physiology.

### Case study 1—population heterogeneity at different growth rates

First, aerobic glucose-limited chemostat processes with different dilution rates were performed to evaluate the influence of growth rate on physiology on population and single cell level. Especially, the relation between the population level growth rate and the expression of EmGFP in combination with *rrnB* should be evaluated. With *E. coli* G7_BL21(DE3)_, chemostats were run at dilution rates of 0.1 h^−1^, 0.2 h^−1^, 0.25 h^−1^, 0.3 h^−1^ and 0.4 h^−1^. Chemostats at the two latter mentioned dilution rates were also run with *E. coli* G5_T7E2_ and additionally at dilution rates of 0.14 h^−1^ and 0.2 h^−1^ (Additional file 3).

### Population level physiology

**Fig. 6:**
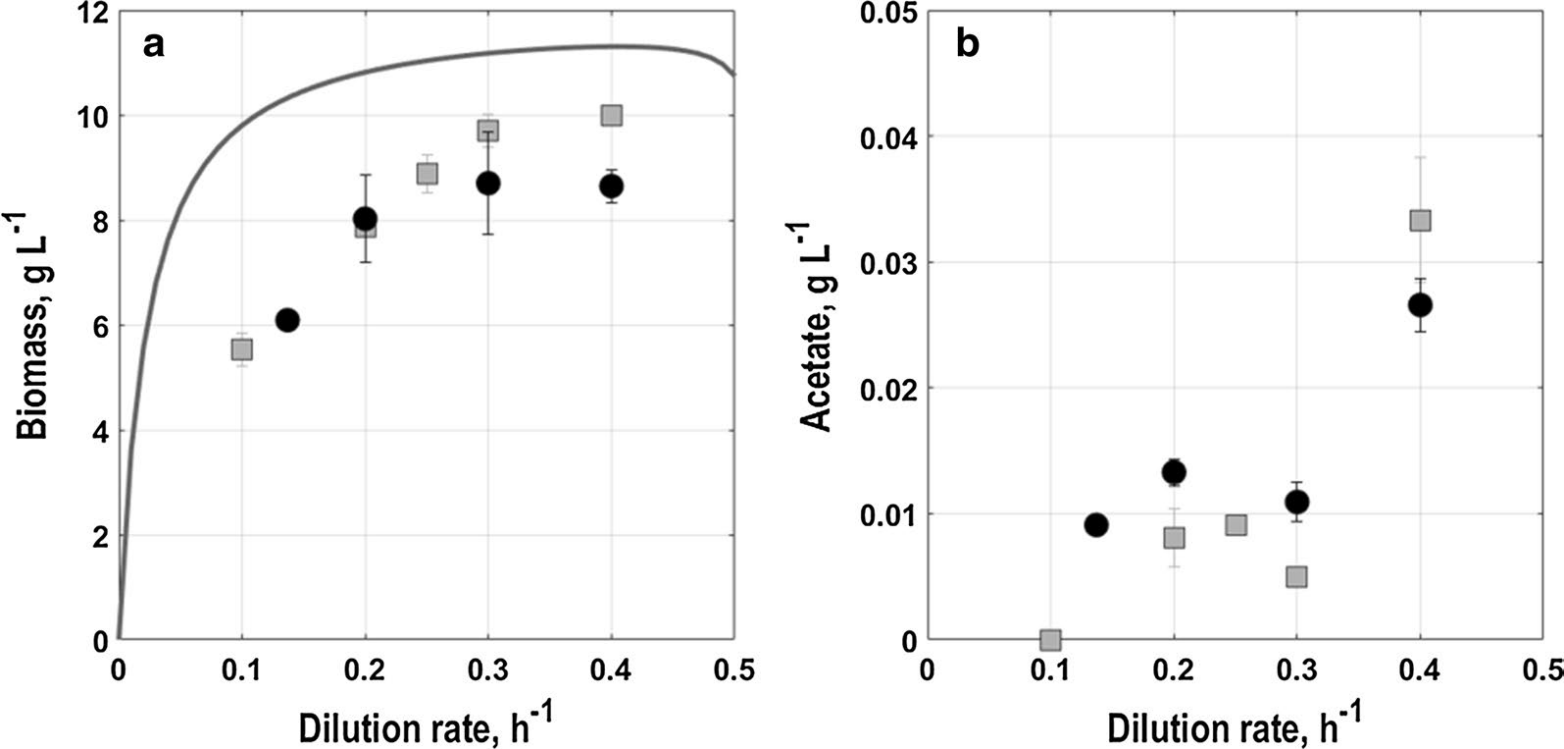
Steady state concentrations of biomass (**a**) and acetate (**b**) in aerobic glucose-limited chemostat cultures with minimal medium in stirred-tank bioreactors (T = 37 °C, pH = 6.8, initial glucose concentration 20 g L^−1^) for the two triple reporter strains *E. coli* G7_BL21(DE3)_ (grey squares) and *E. coli* G5_T7E2_ (black circles). Furthermore, steady state biomass concentrations found under equivalent conditions in continuous cultures with *E. coli* BL21(DE3) carrying the plasmid pET28a-PAmCherry (grey line) are shown as in [59]

Comparing physiology on population level at different dilution rates, the biomass concentration gradually increased by about 45% in cultures with 0.1 h^−1^ until 0.3 h^−1^ for *E. coli* G7_BL21(DE3)_ (Fig. 6a). With further increase of the dilution rate to 0.4 h^−1^, the biomass concentration remained constant. For *E. coli* G5_T7E2_, the trend was similar, but with a lower rise in biomass concentration by about 39% with increasing dilution rate (Fig. 6a). Generally, biomass concentration was slightly lower compared to steady state biomass concentrations found under equivalent conditions in continuous cultures with *E. coli* BL21(DE3) carrying the plasmid pET28a-PAmCherry [59].

For none of the triple reporter strains residual glucose could be detected in the reactor at any of the dilution rates (detection limit of glucose in the HPLC around 0.01 g L^−1^), except at 0.2 h^−1^ for *E. coli* G7_BL21(DE3)_ where minor amounts around 0.01 g L^−1^ were observed (data not shown). Acetate formation as product of overflow metabolism, was only found at dilution rates above 0.3 h^−1^ with both triple reporter strains (Fig. 6b). At 0.3 h^−1^ and beneath only minor amounts of acetate (less than 0.02 g L^−1^) could be measured. At 0.4 h^−1^, acetate started to accumulate in low amounts of around 0.03 g L^−1^. According to published studies (e.g. [59, 60]) the onset of overflow metabolism for *E. coli* lies around 0.45 h^−1^ which is consistent with these results.

### Single cell level physiology

Consistent with population level physiology, on single cell level no expression of *nar*-TagRFP657 respectively oxygen limitation of single cells was detected for any of the dilution rates and none of the two triple reporter strains (Additional file 3). Furthermore, no significant induction of the general stress response of single cells was seen in chemostats at dilution rates of up to and including 0.3 h^−1^ for *E. coli* G7_BL21(DE3)_ and *E. coli* G5_T7E2_. Solely at 0.4 h^−1^ expression of *rpoS*-mStrawberry was slightly increased (10% rise in mean fluorescence intensity compared to negative control, Additional file 3) at the beginning of the continuous phase with *E. coli* G7_BL21(DE3)_, but not with *E. coli* G5_T7E2_. In steady state at 0.4 h^−1^, no significant *rpoS*-mStrawberry expression could be detected with *E. coli* G7_BL21(DE3)_.

**Fig. 7:**
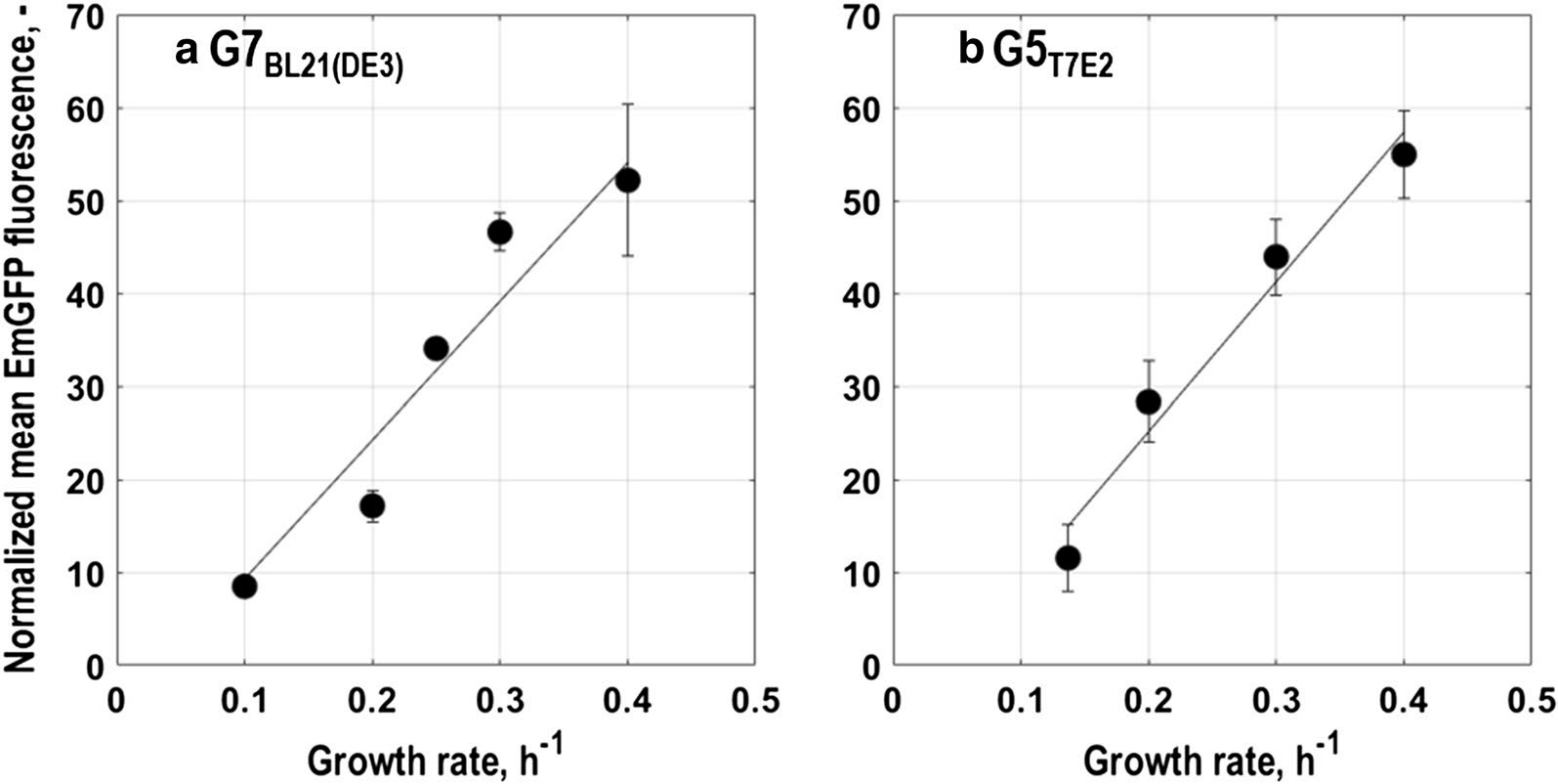
Normalized mean EmGFP fluorescence for single cell growth in aerobic glucose-limited chemostat cultures with minimal medium in stirred-tank bioreactors (T = 37 °C, pH = 6.8, initial glucose concentration 20 g L^−1^) at different dilution rates in steady state normalized by optical density at 600 nm for the triple reporter strains *E. coli* G7_BL21(DE3)_ (**a**) and *E. coli* G5_T7E2_ (**b**). Lines indicate the result of linear regression (coefficient of determination: R^2^ = 0.92 for *E. coli* G7_BL21(DE3)_; R^2^ = 0.97 for *E. coli* G5_T7E2_)

Expression of *rrnB*-EmGFP correlated to single cell growth increased as expected with dilution rate for *E. coli* G7_BL21(DE3)_ and *E. coli* G5_T7E2_. When normalizing mean fluorescence intensity of EmGFP with OD_600_, a linear correlation between normalized mean EmGFP fluorescence and dilution rate was found for both strains (Fig. 7). However, the resulting regression line reveals a 5% better linear fit for the normalized mean fluorescence data of *E. coli* G5_T7E2_ than of *E. coli* G7_BL21(DE3)_ (coefficient of determination R^2^ = 0.97 vs. R^2^ = 0.92). These findings are in agreement with published transcriptomics data and studies at different dilution rates [36, 40].

### Case study 2—population heterogeneity in response to combined oxygen and glucose perturbation

After characterisation of steady state of chemostat cultures at different dilution rates (growth rates), continuous cultures at a dilution rate of 0.2 h^−1^ of both triple reporter strains were perturbed with a 2 g L^−1^ concentrated glucose pulse and simultaneous interruption of aeration for 30 min. In this way, the appearance of substrate gradients in industrial scale bioreactors could be simulated. These lead to formation of zones in the reactor with low oxygen saturation and high substrate availability close to the point of feeding, whereas distant to that, generally opposite conditions are found [61]. Perturbations were followed with frequent sampling for population and single cell level physiology. Thereby, a special focus was put on inducibility of *nar*-TagRFP657 under oxygen limited conditions.

### Population level physiology

Following perturbation on population level, the triple reporter strains exhibited a similar, batch-like behaviour, however produced different amounts of intermediates of the central carbon metabolism.

**Fig. 8:**
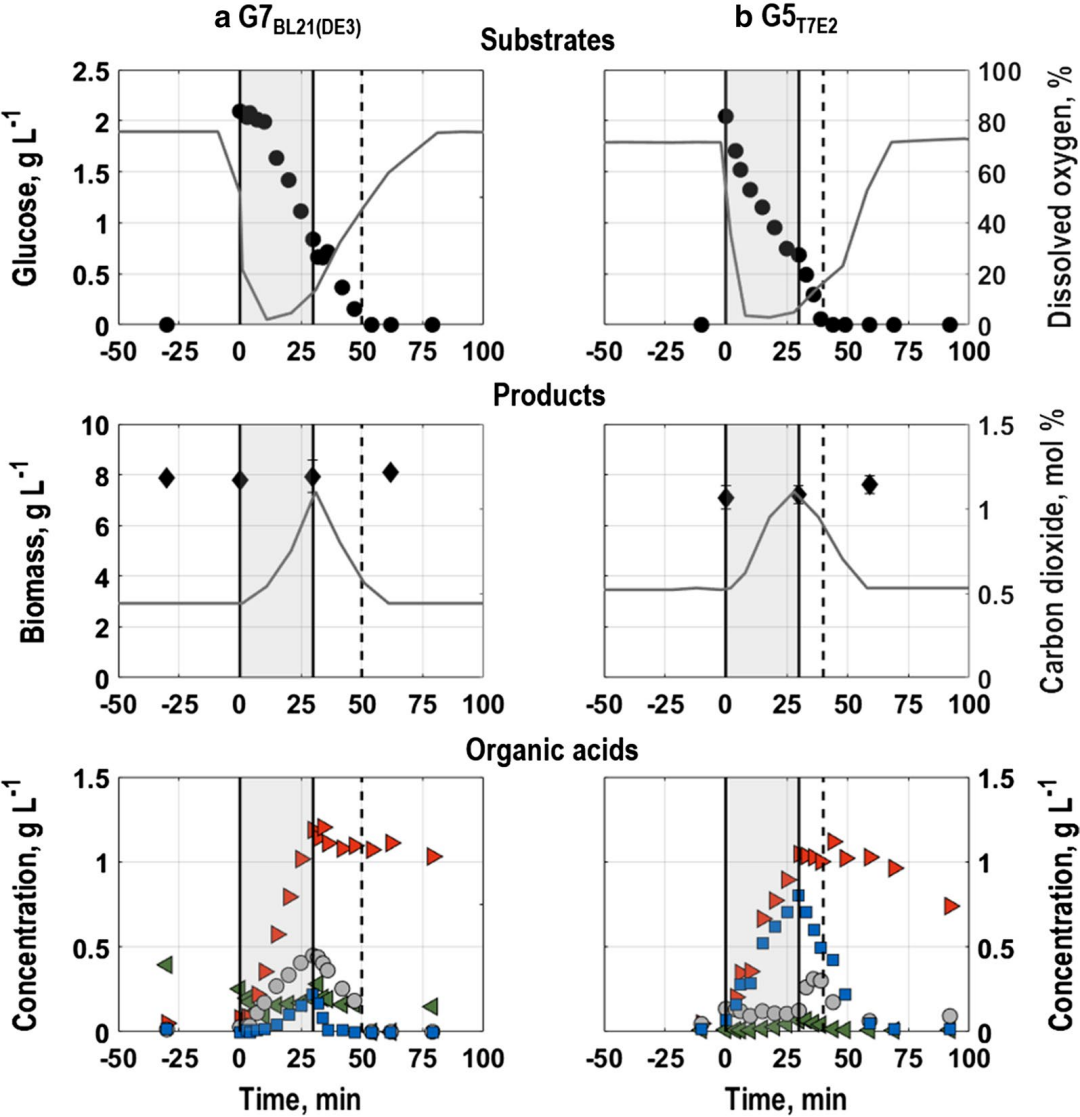
Concentrations of biomass and metabolites [glucose, acetate (blue squares), malate (green left-pointing triangles), formate (red right-pointing triangles) and lactate (grey circles)], off-gas carbon dioxide and dissolved oxygen of the triple reporter strains *E. coli* G7_BL21(DE3)_ (**a**) and *E. coli* G5 _T7E2_ (**b**) following perturbation with 2 g L^−1^ concentrated glucose and simultaneous interruption of air supply for 30 min in aerobic glucose-limited chemostat cultures with minimal medium in stirred-tank bioreactors at 0.2 h^−1^ (T = 37 °C, pH = 6.8, initial glucose concentration 20 g L^−1^). Grey areas illustrate the period without air supply, whereas dashed lines indicate when the pulsed glucose was depleted (around 50 min after perturbation with *E. coli* G7_BL21(DE3)_ and around 45 min after perturbation with *E. coli* G5_T7E2_). Error bars indicate triplicate datasets

For *E. coli* G7_BL21(DE3)_, the response to perturbation can be divided into four phases. Until around 15 min after the perturbation, only minor amounts of the pulsed glucose were consumed (Fig. 8a, substrates). Nevertheless, cells produced formate, lactate and acetate (Fig. 8a, organic acids). Malate, already present in the culture during steady state, was consumed. Simultaneously, the air saturation in the reactor decreased to around 2%, whereas only minor amounts of carbon dioxide were produced (Fig. 8a, substrates and products). Thereafter, until 30 min after perturbation, the pulsed glucose was actively consumed accompanied by a rise in carbon dioxide (Fig. 8a, substrates and products). During this phase, formate, acetate, lactate and minor amounts of malate were also accumulated (Fig. 8a, organic acids). When aeration was restarted, the residual glucose was co-metabolized with the prior produced acetate, malate and lactate. Formate was not re-assimilated and instead gradually washed-out of the reactor (Fig. 8a, organic acids). The dissolved oxygen content in the liquid phase slowly recovered concurrently with decreasing level of carbon dioxide (Fig. 8a, substrates and products). With depletion of the pulsed glucose around 50 min after perturbation, all values returned to steady state from before perturbation, except of formate, which did not return to steady state values within the measurement period (Fig. 8a, organic acids).

In contrast to *E. coli* G7_BL21(DE3)_, *E. coli* G5_T7E2_ started to consume the pulsed glucose directly after perturbation (Fig. 8b, substrates). Simultaneously, acetate and formate were accumulated as well as minor amounts of malate, which was, opposite to the culture with *E. coli* G7_BL21(DE3)_, not present in steady state prior to perturbation (Fig. 8b organic acids). Furthermore, *E. coli* G5_T7E2_ did not acquire any lactate during absence of aeration. While the final concentration of formate was similar to that of *E. coli* G7_BL21(DE3)_ (about 1 g L^−1^), the acetate concentration was around 70% higher. With perturbation, the dissolved oxygen concentration in the liquid phase rapidly decreased to around 1% (Fig. 8b, substrates). During consumption of the pulsed glucose, the off-gas carbon dioxide rose to around 1% (Fig. 8b, products). When aeration was restarted, the prior produced acetate was re-assimilated together with consumption of residual glucose and malate, while low amounts of lactate were produced and shortly after abolished again (Fig. 8b, organic acids). About 45 min after perturbation, the pulsed glucose was depleted, which was faster than for *E. coli* G7_BL21(DE3)_ (Fig. 8a, b, substrates). Afterwards, residual acetate, which was first depleted around 15 min after the pulsed glucose, was re-assimilated, while formate levels remained constant (Fig. 8b, organic acids). Accordingly, carbon dioxide and dissolved oxygen levels in the gas phase only returned slowly to steady state values (Fig. 8b, substrates and products). With depletion of acetate, all values returned to steady state prior to perturbation. Solely formate was slowly washed-out of the reactor and did not return to steady state values within the measurement period.

### Single cell level physiology

#### Single cell growth

**Fig. 9:**
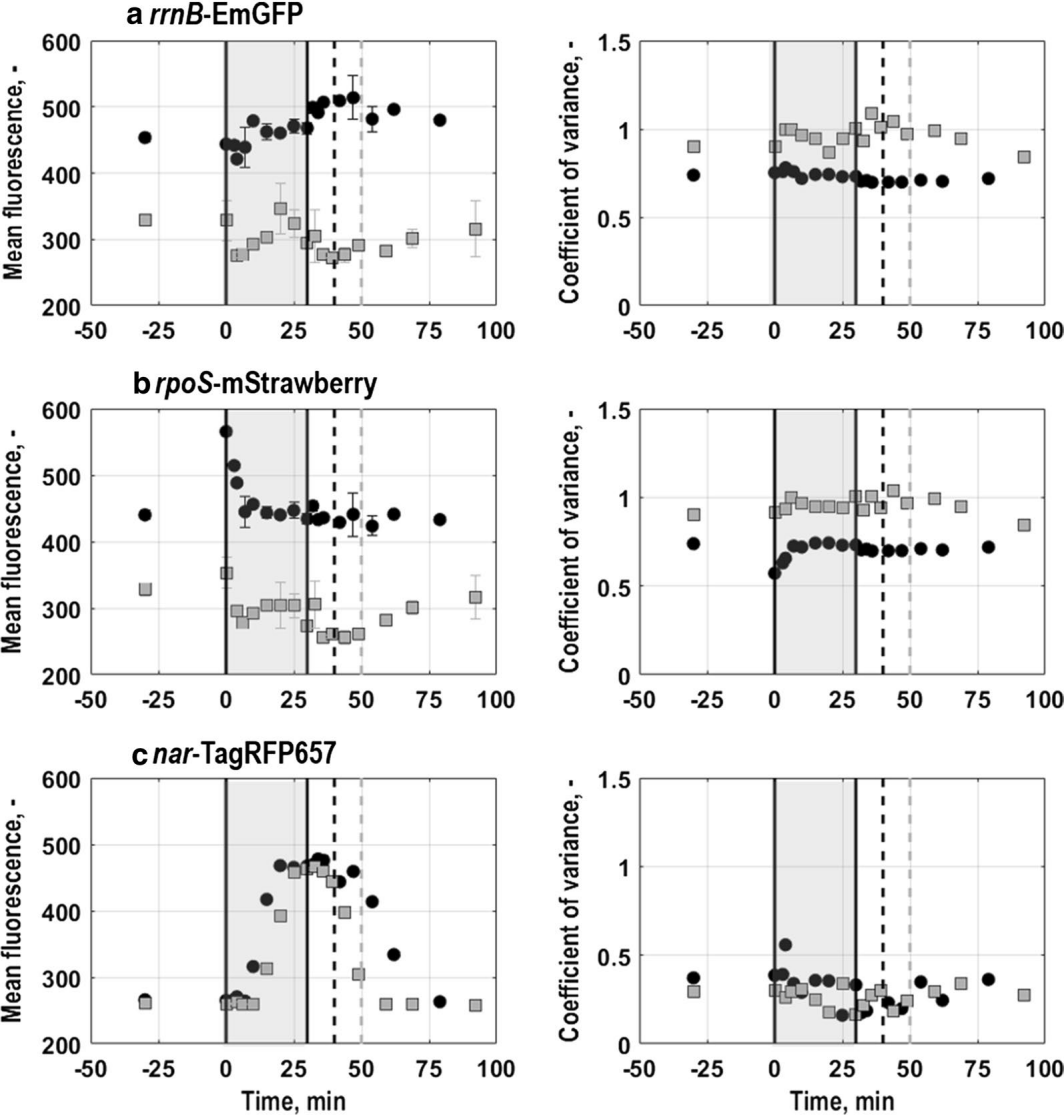
Mean fluorescence (left) and coefficient of variance (right) of the triple reporter strains *E. coli* G7 _BL21(DE3)_ (grey squares) and *E. coli* G5 _T7E2_ (black circles) following perturbation with 2 g L^−1^ concentrated glucose and simultaneous interruption of air supply for 30 min in aerobic glucose-limited chemostat cultures with minimal medium in stirred-tank bioreactors at 0.2 h^−1^ (T = 37 °C, pH = 6.8, initial glucose concentration 20 g L^−1^). Values are shown for single cell growth related to *rrnB*-EmGFP expression (**a**), general stress response of single cells related to *rpoS*-mStrawberry expression (**b**) and oxygen limitation of single cells related to *nar*-TagRFP657 expression (**c**) as average of triplicate measurements. Grey areas illustrate the period without air supply whereas dashed lines indicate when the pulsed glucose was depleted (around 50 min after perturbation with *E. coli* G7_BL21(DE3)_ (grey line) and around 45 min after perturbation with *E. coli* G5_T7E2_ (black line)). Error bars indicate triplicate datasets

The growth of single cells of *E. coli* G7_BL21(DE3)_ was only partly consistent with population level physiology (Figs. 8, 9). Mean EmGFP-fluorescence levels of *E. coli* G7_BL21(DE3)_ slightly fluctuated with perturbation, where after, during consumption of the pulsed glucose (Fig. 8a, substrates), a constant increase in mean EmGFP-fluorescence until around 25 min after perturbation was found (Fig. 9a, Additional file 4). Then, EmGFP-fluorescence levels remained constant apart from a slight deflection when aeration was restarted (Fig. 9a), even though the residual pulsed glucose was further consumed on population level (Fig. 8a, substrates). With depletion of the pulsed glucose around 50 min after perturbation (Fig. 8a, substrates), mean fluorescence intensity returned to steady state levels. Noise in gene expression, depicted as CV of EmGFP-expression (Fig. 9a), slighty changed with perturbation. During glucose consumption, noise in gene expression remained constant, but exhibited a slight deflection when aeration was restarted. Afterwards, variation levels gradually returned to steady state values.

In *E. coli* G5_T7E2_ cultures, mean fluorescence intensity levels for EmGFP-expression (Fig. 9a, Additional file 4) rose following glucose consumption (Fig. 8b, substrates). A constant growth in mean EmGFP fluorescence intensity was seen until aeration was restarted (Fig. 9a). Then, fluorescence intensity increased further until the pulsed glucose was depleted (around 45 min after perturbation, Fig. 8b, substrates). Afterwards, mean EmGFP fluorescence intensity slowly returned to steady state levels prior to perturbation that were reached within around 35 min after depletion of the pulsed glucose (Fig. 9a). Consequently, in contrast to *E. coli* G7_BL21(DE3)_, EmGFP-expression of *E. coli* G5_T7E2_ could mirror growth on population level. Moreover, the CV, which was for *E. coli* G5_T7E2_ generally lower than for *E. coli* G7_BL21(DE3)_, remained almost constant throughout the perturbation (Fig. 9a).

Generally, *E. coli* G5_T7E2_ exhibited higher mean fluorescence levels in conjunction with lower CVs for steady and throughout perturbation than *E. coli* G7_BL21(DE3)_ and could more clearly reflect single cell level growth characteristics described on population level. The prolonged rise in mean fluorescence intensity in cultures with *E. coli* G5_T7E2_ compared to *E. coli* G7_BL21(DE3)_ might be connected to subsequent rather than co-consumption of glucose and acetate, that is accumulated in response to perturbation (Fig. 8a, b, organic acids).

#### General stress response of single cells

General stress response of single cells exhibited the strongest response immediately after perturbation. *E. coli* G7_BL21(DE3)_ showed a non-distinctive response to interruption of aeration and simultaneous introduction of the glucose pulse. With perturbation, a slight fluctuation in mean fluorescence levels could be suspected, that reverted, when the cells started to consume the pulsed glucose (Figs.  8a, 9b, Additional file 4). Thereafter, mean mStrawberry-expression levels remained about constant until aeration was restarted, when mean mStrawberry-expression decreased. With glucose depletion around 50 min after perturbation (8a, substrates), mean fluorescence intensity started to gradually rise towards steady state until the end of the measurement period (Fig. 9b). The CV solely showed light deflections during the perturbation period (Fig. 9b). Other than that, no significant changes were found.

Cells from *E. coli* G5_T7E2_ cultures generally exhibited higher mean fluorescence intensities than cells from cultures with *E. coli* G7_BL21(DE3)_. Furthermore, *E. coli* G5_T7E2_ cultures showed a distinct and steep increase in mean mStrawberry-expression intensity with perturbation (Fig. 9b). However, fluorescence intensity reverted until around 15 min after perturbation to levels prior to perturbation. Afterwards, mStrawberry-fluorescence intensity levels remained about constant until the end of the measurement period. The CV showed the opposite behaviour as mean fluorescence intensity. CV-values decreased compared to steady levels prior to perturbation, but also reverted to steady state values within around 15 min after perturbation (Fig. 9b). Similar to the single cell growth rate, noise levels are generally lower for *E. coli* G5_T7E2_ than for *E. coli* G7_BL21(DE3)_.

#### Oxygen limitation of single cells

Oxygen limitation of single cells showed for both strains distinct changes in fluorescence pattern and -intensity in response to perturbation, which were consistent with population level physiology. Shortly after perturbation, mean TagRFP657-fluorescence levels of both triple reporter strains rose steeply coincident with dissolved oxygen concentration in the liquid phase dropping below 40% air saturation (Figs. 8a, b, substrates, 9c and Additional file 4). Fluorescence levels continued to increase until aeration was restarted, where after they decreased with glucose depletion and relaxation of dissolved oxygen concentration. For *E. coli* G7_BL21(DE3)_, former steady state levels were reached with glucose depletion whereas for *E. coli* G5_T7E2_ steady state levels were first resumed around 60 min after perturbation. The later reversion to steady state values for *E. coli* G5_T7E2_ was probably connected to re-assimilation of residual acetate after glucose depletion.

As the distribution shape seemingly remained constant, the CV is slightly decreasing with increasing TagRFP657-fluorescence levels for *E. coli* G7_BL21(DE3)_ around 15 min after perturbation (Fig. 9c). Lowest noise in gene expression levels were found close to when aeration was restarted, where after the CV returned to steady state values prior to perturbation. Apart from a slight irruption around 15 min after perturbation, CV values for *E. coli* G5_T7E2_ remained constant until aeration was restarted (Fig. 9c). Then, noise in gene expression levels gradually returned to steady state values. Strikingly, in comparison to the other two markers of the *E. coli* triple reporter strains, the CV for TagRFP657 was generally 30-50% lower.

## Discussion

In the present study, two *E. coli* triple reporter strains based on wildtype *E. coli* BL21(DE3) and on a modified version of *E. coli* BL21(DE3), T7E2, have been characterized and evaluated for their suitability for investigation of population heterogeneity in laboratory scale bioprocesses simulating industrial scale process conditions. Both strains could monitor single cell growth through the expression of EmGFP together with the ribosomal promoter *rrnB*, the general stress response of single cells with the expression of *rpoS*-mStrawberry and oxygen limitation of single cells by expression of *nar*-TagRFP657 in different growth phases as well as at different growth rates and in response to perturbations of oxygen and glucose. Furthermore, comparing fluorescence characteristics of the different markers of the triple reporter strains could reveal physiological correlations. Though, the responses of the strains deviated and were not always consistent with population level physiology.

### Differences between the two triple reporter strains

#### Population level physiology

As expected, *E. coli* G7_BL21(DE3)_ exhibited stronger resemblance with *E. coli* BL21(DE3) than with *E. coli* G5_T7E2_ in growth behaviour in batch cultures. However, major characteristics as growth rate, yields and final biomass concentration were similar for the two triple reporter strains. Generally, *E. coli* G5_T7E2_ needed less time to adjust to new environmental conditions, as seen by the shorter lag-phase in batch processes and almost immediate consumption of pulsed glucose in chemostat cultures. Furthermore, the *E. coli* T7E2 based triple reporter strain showed a higher diversity in by-product formation in different phases of the batch as well as in response to the pulse, nevertheless this also resulted in lower steady state biomass concentrations in chemostat cultures at different dilution rates. As mentioned above, the reason could be a higher flexibility in the central carbon metabolism of *E. coli* G5_T7E2_ [45]. Surprisingly, while *E. coli* G7_BL21(DE3)_ co-metabolized acetate and glucose in batch and continuous culture, *E. coli* G5 _T7E2_ rather consumed acetate subsequently to glucose. Possibly, the higher metabolic diversity results in lower priority for acetate re-assimilation, as energy can be restored through other parts of the metabolism [62, 63].

#### Single cell level physiology

Also on single cell level a higher flexibility and responsiveness of *E. coli* G5_T7E2_ was visible as this strain was able to reflect more clearly and sensitively even small changes in population variation and fluorescence intensity in different growth phases in batch cultures, in response to perturbation as well as distinct general stress responses in batch and continuous cultures. Moreover, linearity of normalized mean fluorescence values in continuous culture at different growth rates was higher for *E. coli* G5_T7E2_ than for *E. coli* G7_BL21(DE3)_. Apart from the marker for oxygen limitation in batch cultures, *E. coli* G5_T7E2_ generally exhibited higher mean fluorescence values for all markers under all conditions investigated. At the same time, population variation respectively noise in gene expression was predominantly lower than for *E. coli* G7_BL21(DE3)_ which points towards higher alignment and robustness of *E. coli* G5_T7E2_ cultures as also suspected earlier [45].

Consequently, due to its advantageous physiological characteristics *E. coli* G5_T7E2_ seems to be better suited when population variation in a dynamic bioprocess environment should be monitored. However, *E. coli* G7_BL21(DE3)_ can still be applied to study general trends and moderate changes with slight loss in resolution of physiological changes.

### Suitability of the marker positions and the integrated fluorescence proteins

In general, all utilized markers could reflect the targeted cellular characteristics through their fluorescence distributions collected with flow cytometry in both strains, even though they exhibit slightly different characteristics. While all applied fluorescence proteins were shown to be pH insensitive and are generally monomers, the maturation time of TagRFP657 is higher than for EmGFP and mStrawberry [28, 30].

Probably, therefore *rrnB*-EmGFP and *rpoS*-mStrawberry exhibited, especially considering *E. coli* G5_T7E2_, responses even to small environmental changes as well as an immediate response to perturbation. However, the decay in mean fluorescence when the trigger signal vanishes, was, especially during batch cultures, slow for the signal of both fluorescent proteins. In stationary phase, fluorescence levels remained high until the end of the batch process. Maybe, fluorescence of the reporter proteins is preserved by the conditions that predominate stationary phase cultures of the triple reporter strains, however this assumption would need to be further investigated. Therefore, for futures studies it might be advantageous to add, for instance, a tag to destabilize these two fluorescent proteins to ensure a faster decline in fluorescence signal after its trigger vanishes.

Expression of *rrnB*-EmGFP could be shown to correlate with the growth rate of single cells when normalizing with optical density, whereas just taking raw fluorescence intensity data into account, fluorescence rather correlated to growth on population level. This is possibly connected to the fact that *E. coli* cells increase slightly in length and width in nutrient-rich conditions [64]. In response to glucose perturbation and oxygen limitation only a slight upshift in fluorescence levels was found, which fits with the pulse intensity and the corresponding upshift in growth. However, future studies with a higher pulsed substrate concentration at different growth rates would probably reveal a clearer and stronger response of this marker.

Concerning the general stress response followed with expression of *rpoS*-mStrawberry, it has to be mentioned, that this marker allows to gain a general picture of induction of the stress response. When following batch cultures, the marker exhibited the expected response in mid-/late exponential growth phase. However, during the perturbation with glucose and oxygen limitation the response was rather unspecific, probably due to the fact that cells used specialized stress responses at this point of the process as they are already primed for potential stress respectively encounter generally stable conditions in the chemostat. The latter case would then probably even lead to downregulation of the expression of *rpoS* [41]. This however would need to be addressed in future studies where the targeted induction of the general stress response is investigated in response to stress of different kind. Consequently, for a deeper investigation of the mechanism behind population heterogeneity, the marker probably has to be placed differently in the genome. For instance, a more specific response to a certain kind of stress that is relevant for *E. coli* to cope with environmental changes in industrial scale bioprocesses could be followed.

The expression of TagRFP657 seemed to be slightly sluggish, especially following batch cultures and also expression levels were lower than for the other markers. Moreover, the distribution shape, e.g. in response to oxygen perturbations in continuous cultures seemed to fluctuate, which could not easily be correlated to physiological changes. The reason might be that TagRFP657 has a longer half-life than the other markers [28, 65]. Possibly in the future TagRFP657 could be replaced by a fluorescent protein that exhibits more similar maturation characteristics to mStrawberry and EmGFP.

But also the expression of the *nar*-operon itself might be less stable, as during the present study upshifts in TagRFP657-fluorescence in batch cultures were observed earlier than what was expected from previous studies. There, expression of the *nar*-operon was first induced when oxygen saturation was below 40%, while in the present study expression levels were already increased between 50 and 40% oxygen saturation [37]. However, when oxygen saturation decreased rapidly in response to perturbation, the mean fluorescence response was clear and reverted fast when oxygen saturation in the bioreactor rose again.

### Evaluation of physiological findings correlating the response of the three markers

Throughout this study, single cell physiology was in agreement with or could further supplement population level physiology. In batch cultures, single cell growth could be reflected by EmGFP-expression and exhibited an increase in population variation before the onset of exponential growth phase. Probably, *E. coli* uses noise in gene expression of *rrnB* during lag-phase to prepare for growth, whereas it only plays a minor role during transition from exponential growth to stationary phase, as no such increase was found. Population heterogeneity as a strategy to prepare for growth was also found elsewhere [15, 58]. During exponential growth phase, cells seem to be more aligned than during all other batch phases exhibiting less heterogeneity.

Interestingly, general stress response was induced in mid-exponential growth phase which is consistent with earlier studies [22, 41], but did not lead to a significant increase in noise in gene expression or population heterogeneity. Presumably, the general stress response is a rather conserved function in *E. coli* and therefore not steered by noise in gene expression.

In general for growth rate and general stress response of single cells an inverse correlation between expression and noise in gene expression was observed, which has also been suggested earlier [8]. For oxygen limitation of single cells this correlation was not as clearly visible.

When continuous cultures are simultaneously perturbed with glucose and oxygen, *E. coli* apparently only uses the general stress response to cope with the environmental change immediately after perturbation whereas later, e.g. when the aeration is restarted, cells possibly employ specialized stress responses and re-adjustment mechanisms of the metabolism, respectively, that are not directly correlated to the general stress response. Furthermore, once growing, *E. coli* seems to be prepared to encounter oxygen limited conditions, e.g. when nutrients get scarce as no induction of the general stress response was found simultaneously to expression of the marker for oxygen limitation. However, also specialized stress responses that are automatically primed during growth could be employed.

Expectedly, for single cell growth neither during batch cultures or at different growth rates nor in response to perturbation with oxygen and glucose a subpopulation division in slower and faster growing cells was found for any of the triple reporter strains under the well-mixed conditions studied. Similarly, no diversification appeared during higher growth rates in chemostats after onset of overflow metabolism. This finding is in accordance with previous studies [53–55] and points towards reasonable robustness of *E. coli* BL21(DE3) and *E. coli* T7E2 in response to environmental changes.

Indeed, for future experiments with the triple reporter strains more advanced methods for data treatment for instance algorithms applying flow cytometric fingerprinting [66–69] that have been developed in the past years should be applied. They could potentially reveal further correlations between the changes in expression of the different markers as well as physiological peculiarities under different environmental conditions in bioprocesses. Especially, small changes that are overlooked when just using averaged data or subjective interpretation of fluorescence histograms could reveal new physiological details [70].

## Conclusion

In conclusion, growth rate, general stress response as well as oxygen limitation of single cells of the two triple reporter strains could be detected and the respective markers exhibit the expected fluorescence pattern throughout standard batch cultivations and in response to changes in growth rate and perturbations in glucose and oxygen levels. Consequently, the triple reporter strains represent valuable tools to study population heterogeneity in bioprocesses and supplement population level physiology with less complexity than omics-methods. Correlating the responses of the different markers to each other significantly increases the level of information compared to conventional experiments investigating single cell physiology with single reporter strains when studying population heterogeneity in bioprocesses.

## Supplementary information

**Additional file 1.** Characterization of the triple reporter strains.

**Additional file 2.** Optical density and its correlation with biomass concentration in batch cultures with the triple reporter strains in comparison to the wildtype.

**Additional file 3.** Expression of *rpoS*-mStrawberry and *nar*-TagRFP657 at different growth rates.

**Additional file 4.** Single cell physiology of the triple reporter strains in response to combined perturbation with glucose and oxygen.

## Data Availability

All data generated and analysed during this study are included in this article and its additional files. Raw datasets are available from the corresponding author on reasonable request.
