# Supplementary material for: Development and characterization of Escherichia coli triple reporter strains for investigation of population heterogeneity in bioprocesses
Source: Microb Cell Fact. 2020 Jan 28;19:14. doi: 10.1186/s12934-020-1283-x (PMC6988206; doi:10.1186/s12934-020-1283-x)
Supplement: Supplementary file 1 — Additional file 1. Characterization of the triple reporter strains. [file 12934_2020_1283_MOESM1_ESM.docx]

**Additional File**

**Development and characterization of *Escherichia coli* triple reporter strains for investigation of population heterogeneity in bioprocesses**

Anna-Lena Heins^1^, Jan Reyelt^2^ , Marlen Schmidt^2^, Harald Kranz^2^ , Dirk Weuster-Botz^1^

^1^Technical University of Munich, Institute of Biochemical Engineering, Boltzmannstr. 15, 85748 Garching, Germany

^2^Gene Bridges GmbH, Im Neuenheimer Feld 584, 69120 Heidelberg, Germany

**Additional file 1 - Characterization of the triple reporter strains**

**Investigation of the metabolic burden.** Triplicate shake flask cultures of single-/double- and triple reporter strains (see Table A1) were performed to evaluate whether expression of the fluorescent proteins is a burden to the cells and therefore has an influence on growth. A comparison of the growth rates (Figure A1) illustrates that the growth rates of the modified strain and strains expressing fluorescent proteins do not significantly deviate from the wildtype which was also confirmed by ANOVA (p-value 0.0253). Consequently, expression of the fluorescent proteins does not seem to be a burden to the cells.

**Table S1** Overview of single-, double- and triple reporter strains used in this study

| **Strain** | **Reporter** | **Fluorescent protein** | **Remarks** |
| --- | --- | --- | --- |
| **D_3_** | *rpoS* | mStrawberry | Based on BL21(DE3) |
| **E_5_** | *rpoS*  *nar* | mStrawberry  TagRFP657 |  |
| **G_7_** | *rpoS*  *nar*  *rrnB* | mStrawberry  TagRFP657  EmGFP |  |
| **E_8_** | *rpoS* | mStrawberry | Based on the modified BL21(DE3) strain T7E2 |
| **D_4_** | *rpoS*  *nar* | mStrawberry  TagRFP657 |  |
| **G_5_** | *rpoS*  *nar*  *rrnB* | mStrawberry  TagRFP657  EmGFP |  |


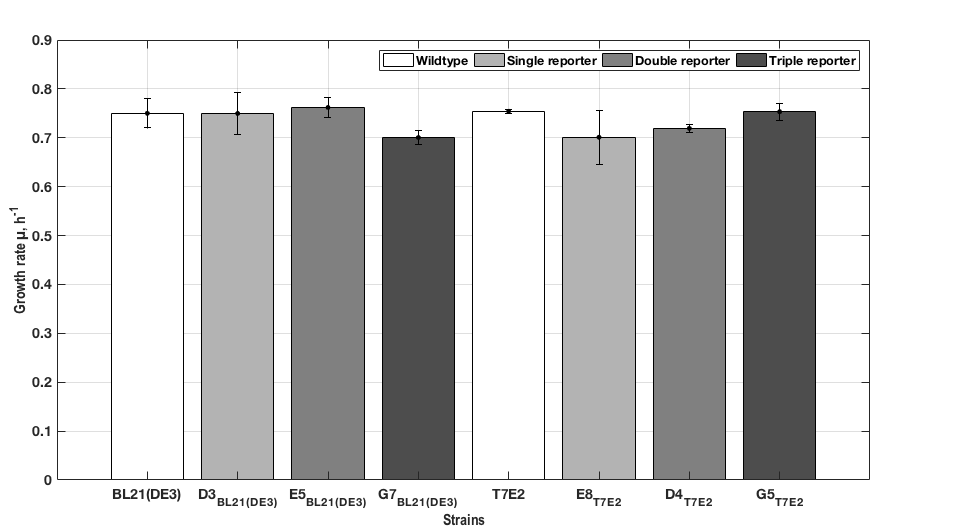


**Figure S1** Comparison of growth rates of single (E8, D3)-, double (E5, D4)- and triple (G5, G7)- reporter strains with wildtype BL21(DE3) and T7E2 grown in Lennox LB medium in shake flasks at 37 °C and shaken at 150 rpm. D3, E5 and G7 are directly based on BL21(DE3) and E8, D4 as well as G5 are based on the modified BL21(DE3) strain T7E2

**Detectability of the fluorescent proteins.** Afterwards, it was investigated whether all fluorescent proteins exhibit a sufficient fluorescence signal that can be detected during a cultivation process. For this purpose conditions were chosen under which the highest fluorescence output was expected for each of the three marker proteins (Figure A2). Samples from mid-exponential growth phase where taken as reference for a bright response in single cell growth whereas late-exponential growth phase was taken as reference for induction of the general stress response and oxygen limitation of single cells. Expression of all fluorescent proteins could be detected under the chosen conditions. Furthermore, reproducibility of distributions was high considering triplicate cultivations (data not shown). For monitoring of general stress response (*rpoS*-mStrawberry) and oxygen limitation of single cells (*nar*-TagRFP657), comparing the two triple reporter strains no significant difference between expression levels respectively fluorescence intensities of the markers could be detected. For single cell growth, G5_T7E2_ exhibited around 25% higher mean fluorescence levels compared to G7_BL21(DE3)_, however fluorescence signals are still sufficiently high. Potentially, G7_BL21(DE3)_ is slightly weaker in its expression machinery because this strain is less streamlined to growth than the modified strain G5 _T7E2_.


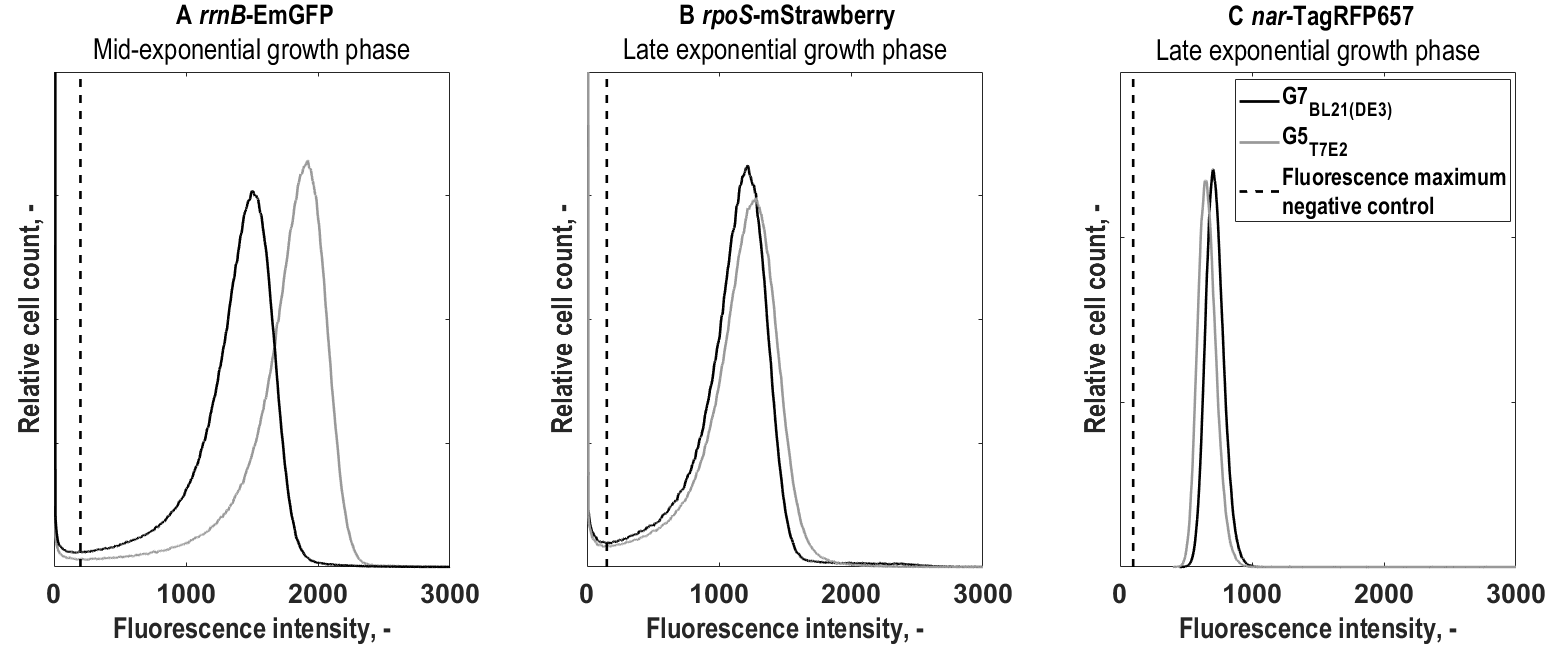


**Figure S2** Fluorescence of the reporter proteins: EmGFP in connection with *rrnB*-expression (A), mStrawberry in connection with expression of *rpoS* (B) and TagRFP657 in connection with *nar*-expression (C) as histogram plots (relative cell count per fluorescence intensity channel of the flow cytometer) for the triple reporter strains G7_BL21(DE3)_ (black) and G5_T7E2_ (grey) grown in shake flask cultures in Lennox LB medium at 37 °C and shaken at 150 rpm. Distributions are shown in relation to negative controls that exhibited no fluorescence of the respective marker. Data shown represent averaged values from biological triplicates

**pH sensitivity of the fluorescent proteins**. After confirming detectability of the fluorescent proteins, it was investigated whether the fluorescence signal is sensitive to pH. Resuspension in buffers of pH 4-8 revealed that fluorescence for mStrawberry and TagRFP657 was insensitive to pH (Figure A3 B and C). Fluorescence of EmGFP exhibited minor variation at different pH. However, these variations were more pronounced at pH values below 5 respectively above 8, so that experiments should be performed at pH 6-7.


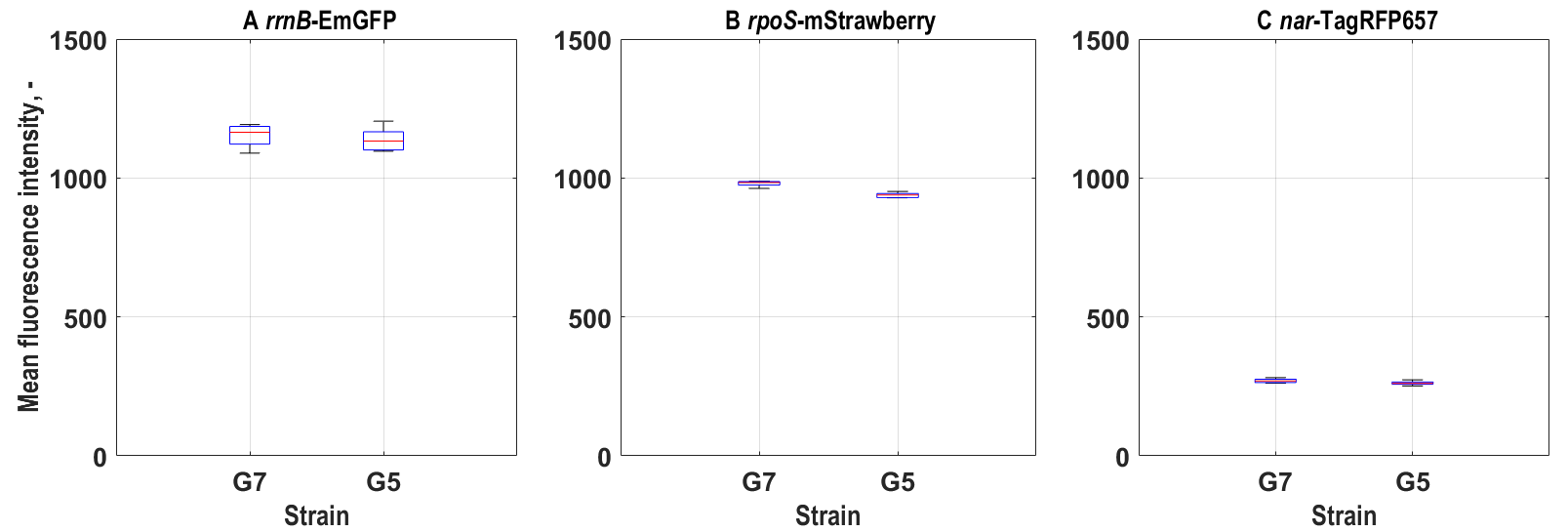


**Figure S3** Boxplots of mean fluorescence intensity of EmGFP *rrnB*-expression (A), mStrawberry in connection with expression of *rpoS* (B) and TagRFP657 in connection with *nar*-expression (C) for both triple reporter strains G7_BL21(DE3)_ (black) and G5_T7E2_ (grey) at pH 4-8 grown in shake flask cultures in Lennox LB medium at 37 °C and shaken at 150 rpm

**Cultivation in shake flask on Lennox LB and minimal medium.** After confirming detectability of fluorescence proteins, shake flask cultures with the two triple reporter strains G7_BL21(DE3)_ and G5_T7E2_ on Lennox LB and minimal medium were performed. Growth was followed with measurement of optical density at 600 nm. Additionally, the resulting fluorescence pattern in single cell growth correlated to *rrnB*-EmGFP expression, general stress response in connection with *rpoS*-mStrawberry expression and the expression of *nar*-TagRFP657 correlated to oxygen limitation, of both strains was investigated with flow cytometry analysis. In both media similar patterns were found, following expression of the three fluorescence proteins throughout the cultivations. However, seemingly fluorescence levels are slightly less condensed in LB medium compared to minimal medium, though the by the characteristics of the fluorescence proteins expected pattern can still be observed.


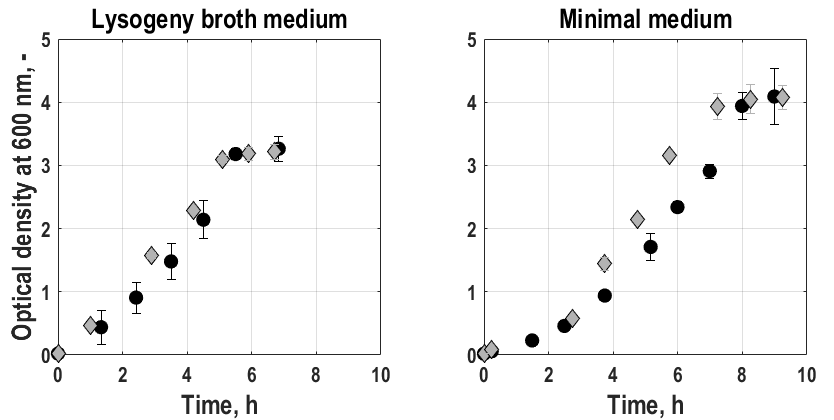


**Figure S4** Optical density at 600 nm for the triple reporter strains *E. coli* G5_T7E2_ (diamonds) and *E. coli* G7_BL21(DE3)_ (circles) following batch cultures in shake flasks for growth on lysogeny broth (LB)- and minimal medium according to Riesenberg (47) (T = 37 °C and 150 rpm). Error bars indicate biological triplicates


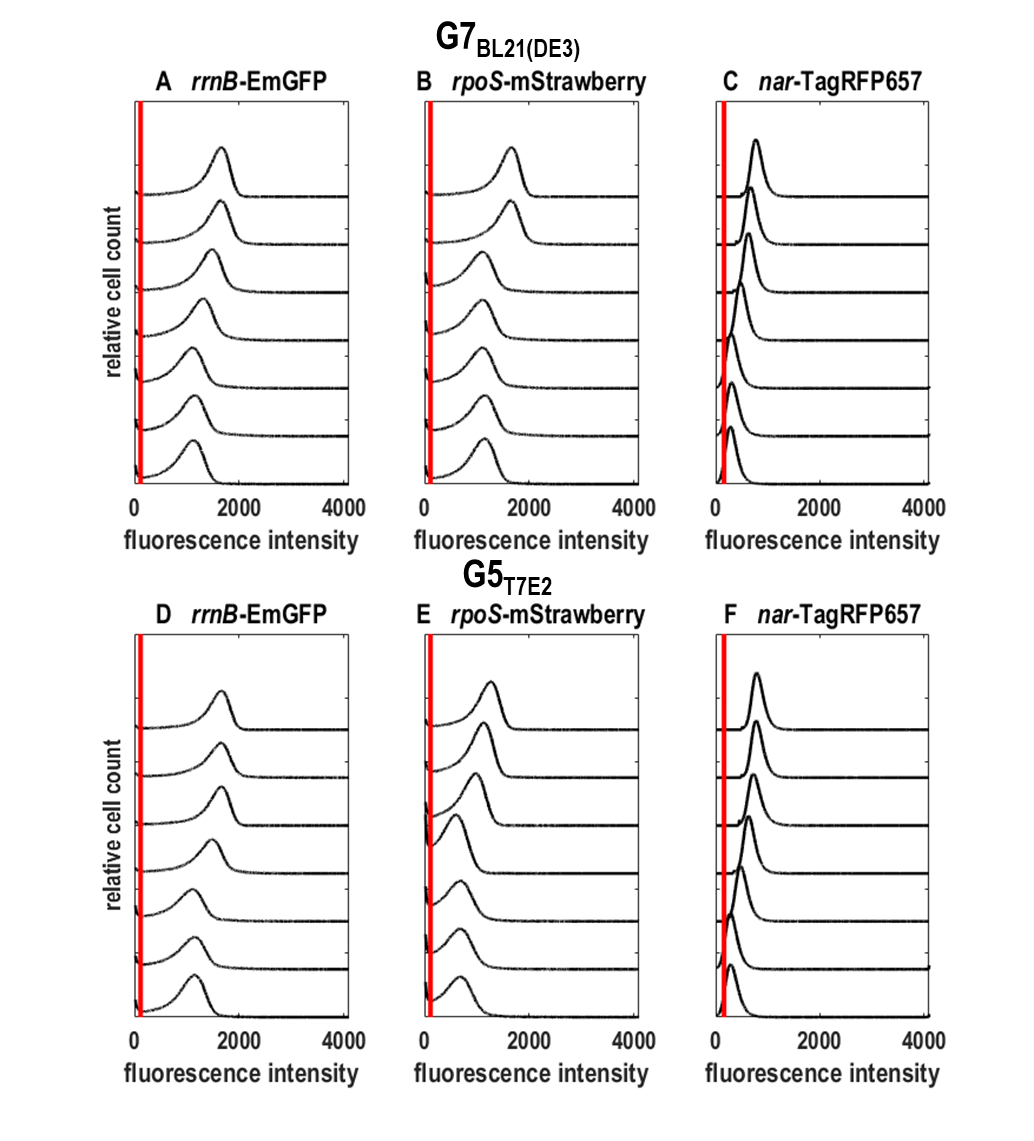


**Figure S5** Single cell physiology for the triple reporter strains *E. coli* G7_BL21(DE3)_ (A-C) and *E. coli* G5_T7E2_ (D-F) following batch cultures in shake flasks for growth on lysogeny broth (LB) (T = 37 °C and 150 rpm). Single cell growth is followed by *rrnB*-EmGFP expression, general stress response of single cells is related to *rpoS*-mStrawberry expression and oxygen limitation of single cells which is followed by *nar*-TagRFP657 expression. Data shown represent averaged values from biological triplicates. Distributions are shown in relation to negative controls that exhibited no fluorescence of the respective marker


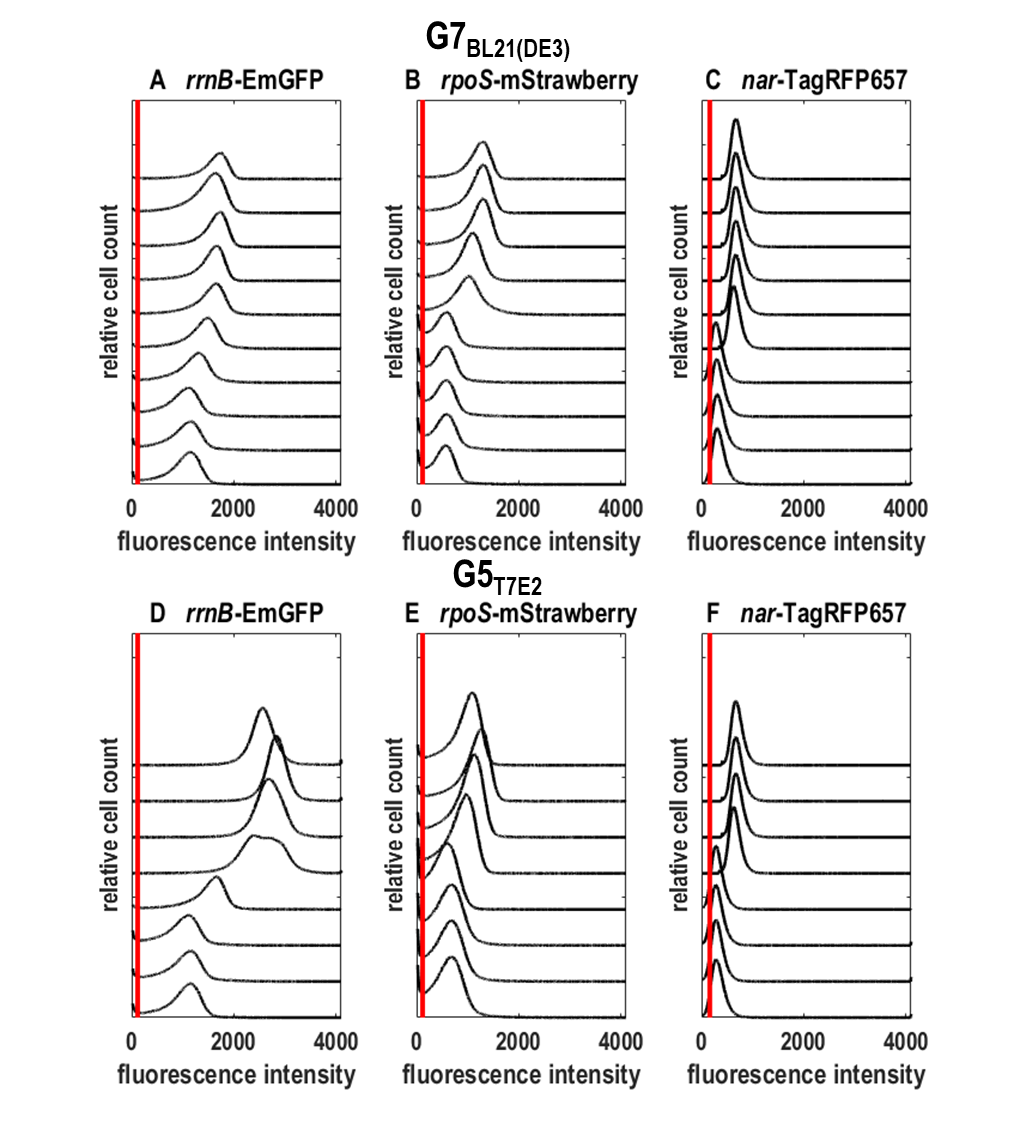


**Figure S6** Single cell physiology for the triple reporter strains *E. coli* G7_BL21(DE3)_ (A-C) and *E. coli* G5_T7E2_ (D-F) following batch cultures in shake flasks for growth on minimal medium according to Riesenberg (47) (T = 37 °C and 150 rpm). Single cell growth is followed by *rrnB*-EmGFP expression, general stress response of single cells is related to *rpoS*-mStrawberry expression and oxygen limitation of single cells which is followed by *nar*-TagRFP657 expression. Data shown represent averaged values from biological triplicates. Distributions are shown in relation to negative controls that exhibited no fluorescence of the respective marker

**Materials and Methods S1**

**Shake flask cultures**

**Pre-cultures**. Single colonies from Lennox Lysogeny Broth (LB) or minimal medium agar plates of the single-, double- and triple-reporter strains respectively BL21(DE3) and T7E2 were used to inoculate 50 mL Lennox LB or minimal medium (according to (Riesenberg, Schulz et al. 1991)) cultures in 500 mL baffled shake flasks as pre-cultures. The flasks were shaken overnight (around 6 h) at 150 rpm and 37 °C and then used to inoculate the main culture.

**Shake flask cultures**. The amount of pre-culture necessary to achieve an optical density OD_600_ of 0.02 was used to inoculate another 500 mL baffled shake flask with 100 mL Lennox LB or minimal medium. The main culture was incubated under the same conditions as the pre-culture and followed by frequent sampling for OD_600_ and were applicable flow cytometry analysis until the cells reached stationary phase. Additionally, for the triple reporter strains and BL21(DE3) (negative control), samples for flow cytometry (FC) analysis were taken in mid- and late exponential growth phase and analysed directly. To ensure comparability between strains, samples were withdrawn at the same time point in the respective growth phases. All experiments were performed in triplicate so that variation between samples is considered by error bars.

**Investigation of pH sensitivity.** Samples taken from shake flask cultures were re-suspended in buffers of pH 4-8. Afterwards the samples were analysed with flow cytometry.

**Sequence of the *rpoS* promoter.** Because several regulatory elements are found upstream of the promoter the promoter sequence, which is however not interrupted by the integrated fluorescence protein, is provided below.

Sequence of the promoter


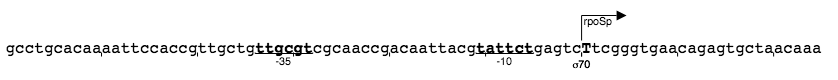


Sequence of two CRP-cAMP DNA-binding transcriptional dual regulators that repress transcription


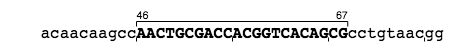


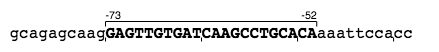


Sequence of two phosporylated DNA-binding transcriptional dual regulatores ArcA that repress transcription


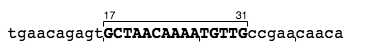


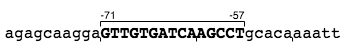


**References**

Riesenberg, D., V. Schulz, W. A. Knorre, H.-D. Pohl, D. Korz, E. A. Sanders, A. Roß and W.-D. Deckwer (1991). "High cell density cultivation of *Escherichia coli* at controlled specific growth rate." Journal of biotechnology **20**: 17-28.
