# Supplementary material for: Development and characterization of Escherichia coli triple reporter strains for investigation of population heterogeneity in bioprocesses
Source: Microb Cell Fact. 2020 Jan 28;19:14. doi: 10.1186/s12934-020-1283-x (PMC6988206; doi:10.1186/s12934-020-1283-x)
Supplement: Supplementary file 2 — Additional file 2. Optical density and its correlation with biomass concentration in batch cultures with the triple reporter strains in comparison to the wildtype. [file 12934_2020_1283_MOESM2_ESM.docx]

**Additional File**

**Development and characterization of *Escherichia coli* triple reporter strains for investigation of population heterogeneity in bioprocesses**

Anna-Lena Heins^1^, Jan Reyelt^2^ , Marlen Schmidt^2^, Harald Kranz^2^ , Dirk Weuster-Botz^1^

^1^Technical University of Munich, Institute of Biochemical Engineering, Boltzmannstr. 15, 85748 Garching, Germany

^2^Gene Bridges GmbH, Im Neuenheimer Feld 584, 69120 Heidelberg, Germany

**Additional File 2 – Optical density and its correlation with biomass concentration in batch cultures with the triple reporter strains in comparison to the wildtype**

**
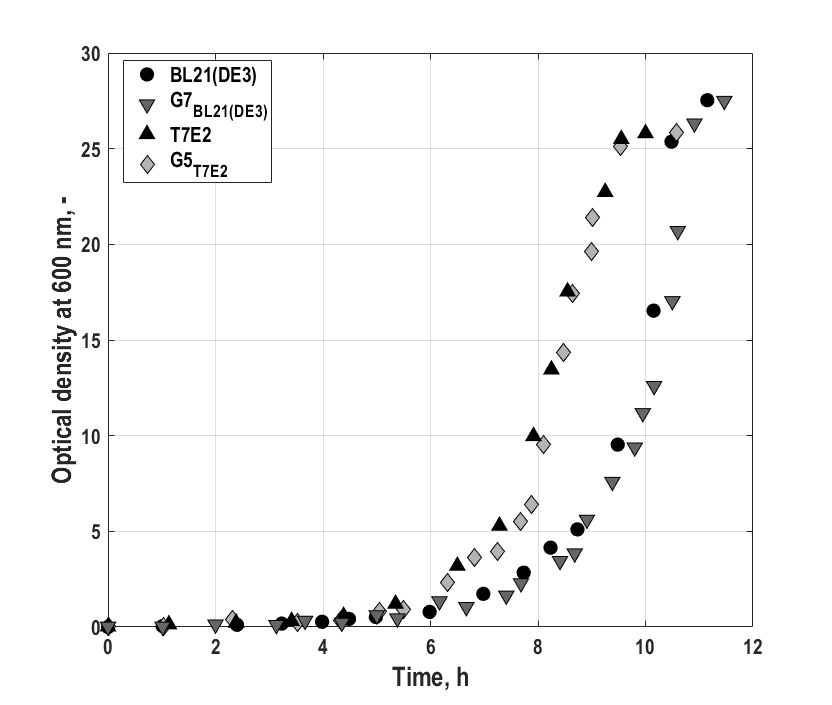
**

**Figure S7** Optical density at 600 nm for the triple reporter strains *E. coli* G5_T7E2_ (diamonds) and *E. coli* G7_BL21(DE3)_ (downwards pointing triangles) following batch processes in stirred-tank bioreactors in comparison to the wildtypes *E. coli* BL21(DE3) (circles) and *E. coli* T7E2 (upwards pointing triangles) for growth on minimal medium according to Riesenberg (47) (T = 37 °C, pH = 6.8, initial glucose concentration 20 g L^-1^).

During exponential growth, correlations between OD_600_ and biomass concentration (DW) could be established (for data for biomass concentration consult main manuscript):

*E. coli* BL21(DE3): 0.497*OD_600_ = DW; R^2^ = 0.99 [ Equation 1]

*E. coli* G7_BL21(DE3)_: 0.491*OD_600_ = DW; R^2^ = 0.98 [Equation 2]

*E. coli* T7E2: 0.483*OD_600_ = DW; R^2^ = 0.98 [Equation 3]

*E. coli* G5_T7E2_: 0.487*OD_600_ = DW; R^2^ = 0.99 [Equation 4]

**References**

Riesenberg, D., V. Schulz, W. A. Knorre, H.-D. Pohl, D. Korz, E. A. Sanders, A. Roß and W.-D. Deckwer (1991). "High cell density cultivation of *Escherichia coli* at controlled specific growth rate." Journal of biotechnology **20**: 17-28.
