# Supplementary material for: Development and characterization of Escherichia coli triple reporter strains for investigation of population heterogeneity in bioprocesses
Source: Microb Cell Fact. 2020 Jan 28;19:14. doi: 10.1186/s12934-020-1283-x (PMC6988206; doi:10.1186/s12934-020-1283-x)
Supplement: Supplementary file 3 — Additional file 3. Expression of rpoS-mStrawberry and nar-TagRFP657 at different growth rates. [file 12934_2020_1283_MOESM3_ESM.docx]

**Additional File**

**Development and characterization of *Escherichia coli* triple reporter strains for investigation of population heterogeneity in bioprocesses**

Anna-Lena Heins^1^, Jan Reyelt^2^ , Marlen Schmidt^2^, Harald Kranz^2^ , Dirk Weuster-Botz^1^

^1^Technical University of Munich, Institute of Biochemical Engineering, Boltzmannstr. 15, 85748 Garching, Germany

^2^Gene Bridges GmbH, Im Neuenheimer Feld 584, 69120 Heidelberg, Germany

**Additional File 3 – Expression of *rpoS*-mStrawberry and *nar*-TagRFP657 at different growth rates**

| **G7_BL21(DE3)_** | **Mean fluorescence intensity** | |
| --- | --- | --- |
|  | ***rpoS*-mStrawberry** | ***nar*-TagRFP657** |
| **0.10** | 120.44 ± 15.23 | 150.45 ± 16.61 |
| **0.20** | 116.34 ± 16.32 | 174.32 ± 20.35 |
| **0.25** | 118.32 ± 23.81 | 166.77 ± 31.35 |
| **0.30** | 116.87 ± 20.12 | 173.53 ± 23.84 |
| **0.40** | 115.65 ± 17.31 | 157.33 ± 13.93 |
| **Negative control** | 116.87 ± 27.21 | 164.24 ± 12.73 |

| **G5_T7E2_** | **Mean fluorescence intensity** | |
| --- | --- | --- |
|  | ***rpoS*-mStrawberry** | ***nar*-TagRFP657** |
| **0.125** | 110.92 ± 23.83 | 155.43 ± 35.72 |
| **0.20** | 111.13 ± 15.87 | 145.98 ± 17.94 |
| **0.30** | 115.23 ± 16.74 | 162.32 ± 16.84 |
| **0.40** | 115.57 ± 34.01 | 171.69 ± 37.77 |
| **Negative control** | 116.25 ± 23.17 | 162.51 ± 19.51 |

**References**

Riesenberg, D., V. Schulz, W. A. Knorre, H.-D. Pohl, D. Korz, E. A. Sanders, A. Roß and W.-D. Deckwer (1991). "High cell density cultivation of *Escherichia coli* at controlled specific growth rate." Journal of biotechnology **20**: 17-28.
