# Supplementary material for: Development and characterization of Escherichia coli triple reporter strains for investigation of population heterogeneity in bioprocesses
Source: Microb Cell Fact. 2020 Jan 28;19:14. doi: 10.1186/s12934-020-1283-x (PMC6988206; doi:10.1186/s12934-020-1283-x)
Supplement: Supplementary file 4 — Additional file 4. Single cell physiology of the triple reporter strains in response to combined perturbation with glucose and oxygen. [file 12934_2020_1283_MOESM4_ESM.docx]

**Additional File**

**Development and characterization of *Escherichia coli* triple reporter strains for investigation of population heterogeneity in bioprocesses**

Anna-Lena Heins^1^, Jan Reyelt^2^ , Marlen Schmidt^2^, Harald Kranz^2^ , Dirk Weuster-Botz^1^

^1^Technical University of Munich, Institute of Biochemical Engineering, Boltzmannstr. 15, 85748 Garching, Germany

^2^Gene Bridges GmbH, Im Neuenheimer Feld 584, 69120 Heidelberg, Germany

**Additional File 4 – Single cell physiology of the triple reporter strains in response to combined perturbation with glucose and oxygen**


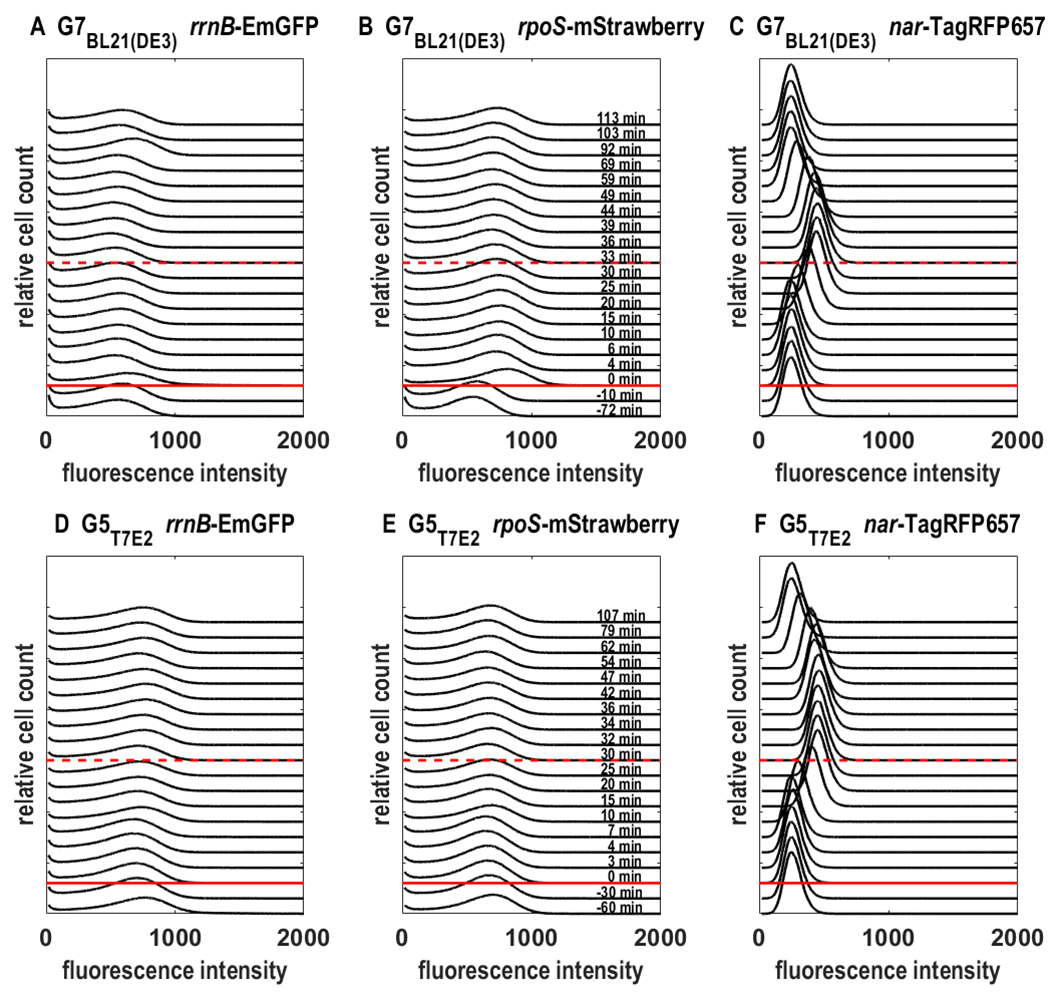


**Figure S8** Single cell level physiology for the triple reporter strains G7 _BL21(DE3)_ (A, B, C) and G5 _T7E2_ (D, E, F) in aerobic glucose-limited chemostat cultures with minimal medium according to Riesenberg (47) (T = 37 °C, pH = 6.8, initial glucose concentration 20 g L^-1^) at 0.2 h^-1^ perturbed with 2 g L^-1^ concentrated glucose pulse and simultaneous interruption of oxygen supply for 30 min. Fluorescence distributions for single cell growth related to *rrnB*-EmGFP expression (A, D), general stress response of single cells related to *rpoS*-mStrawberry expression (B, E) and oxygen limitation of single cells related to *nar*-TagRFP657 (C, F) expression are depicted as average of triplicate measurements. Red full line indicate the pulse start and dashed lines indicate the time when aeration was re-started

**References**

Riesenberg, D., V. Schulz, W. A. Knorre, H.-D. Pohl, D. Korz, E. A. Sanders, A. Roß and W.-D. Deckwer (1991). "High cell density cultivation of *Escherichia coli* at controlled specific growth rate." Journal of biotechnology **20**: 17-28.
